# Acidic amino acids as counterions of ciprofloxacin: Effect on growth and pigment production in *Staphylococcus aureus* NCTC 8325 and *Pseudomonas aeruginosa* PAO1

**DOI:** 10.1371/journal.pone.0250705

**Published:** 2021-04-29

**Authors:** Annsar Ahmad Warraich, Afzal Ur Rahman Mohammed, Hazel Gibson, Majad Hussain, Ayesha Sabah Rahman

**Affiliations:** 1 Aston Pharmacy School, Aston University, Birmingham, United Kingdom; 2 School of Pharmacy, University of Wolverhampton, Wolverhampton, United Kingdom; 3 Quest Healthcare Ltd, Birmingham, United Kingdom; Nitte University, INDIA

## Abstract

Antimicrobial resistance (AMR) is emerging as a global threat to public health. One of the strategies employed to combat AMR is the use of adjuvants which act to enhance or reinstate antimicrobial activity by inhibiting resistance mechanisms. However, these adjuvants are themselves not immune to selecting resistant phenotypes. Thus, there is a need to utilise mechanisms which are either less likely to or unable to trigger resistance. One commonly employed mechanism of resistance by microorganisms is to prevent antimicrobial uptake or efflux the antibiotic which manages to permeate its membrane. Here we propose amino acids as antimicrobial adjuvants that may be utilizing alternate mechanisms to fight AMR. We used a modified ethidium bromide (EtBr) efflux assay to determine its efflux in the presence of ciprofloxacin within Staphylococcus aureus (NCTC 8325) and Pseudomonas aeruginosa (PAO1). In this study, aspartic acid and glutamic acid were found to inhibit growth of both bacterial species. Moreover, a reduced production of toxic pigments, pyocyanin and pyoverdine by *P*. *aeruginosa* was also observed. As evident from similar findings with tetracycline, these adjuvants, may be a way forward towards tackling antimicrobial resistance.

## 1. Introduction

Amino acids (AA) are versatile molecules which have a multitude of functions and can be used as anti-biofilm agents, drug excipients, drug solubility enhancers, and drug adjuvants [1]. Previously, it has been shown that both D- and L-isoforms of acidic amino acids are able to exhibit anti-biofilm activity on their own and act synergistically with Ciprofloxacin (Cip) [2]. The current study investigates whether these counterions, L-aspartic acid (L-Asp) and L-glutamic acid (L-Glu), also possess intrinsic anti-microbial activity on planktonic bacterial cells and if co-treatment with ciprofloxacin leads to further inhibition of planktonic growth. Apart from the aforementioned reason, Cip was chosen as it has been demonstrated in the past that its solubility and permeability can be enhanced using acidic L-amino acids [3,4].

Being active against both, Gram-negative and Gram-positive bacteria, Cip is often a widely used antibiotic for post-procedural prophylaxis [5,6]. It is also often a drug of choice for many infections ranging from eye and ear infections to respiratory tract and urinary tract infections, as indicated by the British National Formulary [6,7]. Cip belongs to the fluoroquinolone class of antibiotics and is bactericidal in effect. Its mechanism of action involves interference with DNA replication by inhibiting DNA-gyrase and DNA topoisomerase activity [8].

As with many other antibiotics, the future use of ciprofloxacin is under threat. Resistance to Cip is on an ever-increasing global rise [9,10]. Three main routes of resistance to Cip are discussed in the literature. Firstly, mutations in the enzymes, DNA-gyrase and DNA topoisomerase IV render the drug unable to act on these targets, allowing DNA replication to function normally. Another mechanism of resistance involves the restriction of Cip accumulation within the bacteria. Bacteria can restrict Cip accumulation by either restricting the amount of Cip that can penetrate into it and/or increasing its efflux. Finally, bacteria can acquire resistance to Cip through plasmid-located genes [11,12]. Considering the role of amino acids in enhancing permeability of various drugs in Caco-2 monolayers as well as buccal cell layers, the second mechanism is of most interest here [3,13].

The route of uptake of Cip is certainly different between Gram-positive and Gram-negative bacteria, and can be attributed to the presence of an outer membrane in Gram-negative bacteria. Whilst Cip most probably penetrates the membrane of Gram-positive bacteria through passive diffusion, the mechanism needed for penetration into Gram-negative bacteria is a little more complicated. Briefly, the asymmetric nature of the outer membrane in Gram-negative bacteria makes it rigid and thus more resistant to passive diffusion of hydrophobic compounds [14]. Due to this, drugs like Cip pass through the outer membrane using porins [12]. Also, Gram-negative bacteria can confer resistance by altering expression of these membrane proteins, limiting uptake of the drug [11].

Various efflux pumps have been identified to extrude fluoroquinolones in several bacteria. In *P*. *aeruginosa*, the majority of pumps belong to the RND superfamily and include MexAB-OprM, MexCD-OprJ, MexEF-OprN and MexXY. Whereas, the MFS superfamily makes up most of the efflux identified currently in *S*. *aureus* and includes NorA, NorB, NorC and SdrM [11].

Thus, this highlights a mechanism which can be used to combat antimicrobial resistance (AMR), i.e. enhancing drug accumulation by increasing penetration of the drug through the membrane/s and/or hindering the efflux of the drug. These mechanisms are of particular interest here because as mentioned earlier, amino acids have been shown to enhance drug permeation into cells. This paper thus studies whether amino acids can combat AMR and proposes the potential ways this could be occurring. For now, as a first step, the effect of amino acids and their combinations with Cip on the efflux of ethidium bromide (EtBr) was studied.

To investigate this, the effect of acidic amino acids on their own and in combination with Cip on planktonic bacteria was studied. It was decided to use the strains *S*. *aureus* (NCTC 8325) and *P*. *aeruginosa* (PA01). This is crucial as these pathogens are among the priority pathogens which the WHO listed in a bid to help focus research and development [15] to combat AMR. To add to this, using these bacteria also allowed the investigation of antimicrobial properties of L-Asp and L-Glu in both Gram-positive (*S*. *aureus*) and Gram-negative (*P*. *aeruginosa*) bacteria.

The study evaluated whether amino acids are able to enhance accumulation of EtBr within planktonic bacterial cells and whether this results in increased antimicrobial activity of the drug. For this, we investigated the use of acidic amino acids as counterions to Cip and determined the effect on the accumulation of EtBr in planktonic *S*. *aureus* and *P*. *aeruginosa* cells. The effect of efflux competitors on the efflux of Nile Red has been conducted in the past using efflux assays [16]. Here we employ similar methods, utilizing another commonly used dye, EtBr, to determine its accumulation in the presence of amino acids and their combinations with Cip as potential efflux competitors [17]. Along with this, we further evaluated any enhancement in antimicrobial activity, including the effect on bacterial growth in both *S*. *aureus* and *P*. *aeruginosa* and pigment production in *P*. *aeruginosa*, with the use of these combinations.

## 2. Results

### 2.1 Minimum inhibitory concentrations (MICs) and minimum bactericidal concentrations (MBCs) of ciprofloxacin

Before conducting accumulation experiments, MICs and MBCs of the amino acids and Cip were conducted on the *S*. *aureus strain* NCTC 8325 and *P*. *aeruginosa* strain PAO1. Concentrations of amino acids screened for MICs for each bacteria were 30, 15, 7.5, 3.75, 1.88, 0.94, 0.47, 0.23 mM whilst the concentrations of Cip screened were 0.14, 0.068, 0.034, 0.017, 0.0085, 0.0042 and 0.0021 mM. All amino acid concentrations tested were below the MICs. Working concentrations of the drug used for EtBr accumulation studies were all below the MICs determined, as presented in Table 1.

**Table 1:** Determination of minimum inhibitory concentrations (MICs) and minimum bactericidal concentrations (MBCs) of ciprofloxacin.

	MIC	MBC
*S*. *aureus* NCTC 8325	5.63 mg/L (16.98 μM)	11.25 mg/L (33.95 μM)
*P*. *aeruginosa* PAO1	0.70 mg/L (2.12 μM)	1.40 mg/L (4.24 μM)

### 2.2 Efflux assessment in *S*. *aureus*

A slightly modified assay was used for efflux assessment of EtBr [18]. This provides insight into the effect of amino acids and their combinations with Cip on the efflux behavior of EtBr. It is possible that this may indicate the efflux behaviour of Cip, however for this will need to be confirmed with further studies directly assessing accumulation of Cip. The experiment was conducted in two parts. The first part was for a duration of 60 min in which the bacteria were deprived of energy (glucose) required for efflux. This was achieved by centrifugation and removal of supernatant. The cells were then loaded with maximum EtBr. The second part of the experiment was conducted for 120 min and was the main part used to assess whether the test substances used were able to effect accumulation or efflux of EtBr. The concentrations of Cip used in the assessment of efflux of EtBr in *S*. *aureus* were 8.49 μM, 4.24 μM, 2.12 μM and 1.06 μM. The amino acids investigated for potential modulation of Cip efflux were L-Asp and L-Glu. To do this, each of the above mentioned Cip concentration were studied individually with L-Asp and L-Glu concentrations of 30 Mm,15 mM, 7.5 mM, 3.75 mM, 1.88 mM, 0.94 mM, 0.47 mM and 0.23 mM.

Along with the test compounds above, various controls were also used in a bid to better understand the observed patterns of efflux. The ‘No Drug’ control consisted of 30 mM L-Asp or L-Glu on its own and was used to gain insight into the effect of the amino acid on efflux of EtBr whilst EtBr had no competition for the efflux pump from Cip. On the other hand, the control ‘0 mM L-Asp or 0 mM L-Glu’ which contained Cip on its own was essential in determining whether Cip modulated EtBr efflux or not. It was also necessary to assess the efflux of EtBr in the presence (glu) and absence (no glu) of glucose which is the energy source.

#### 2.2.1 Efflux of EtBr in *S*. *aureus*

**Fig 1:**
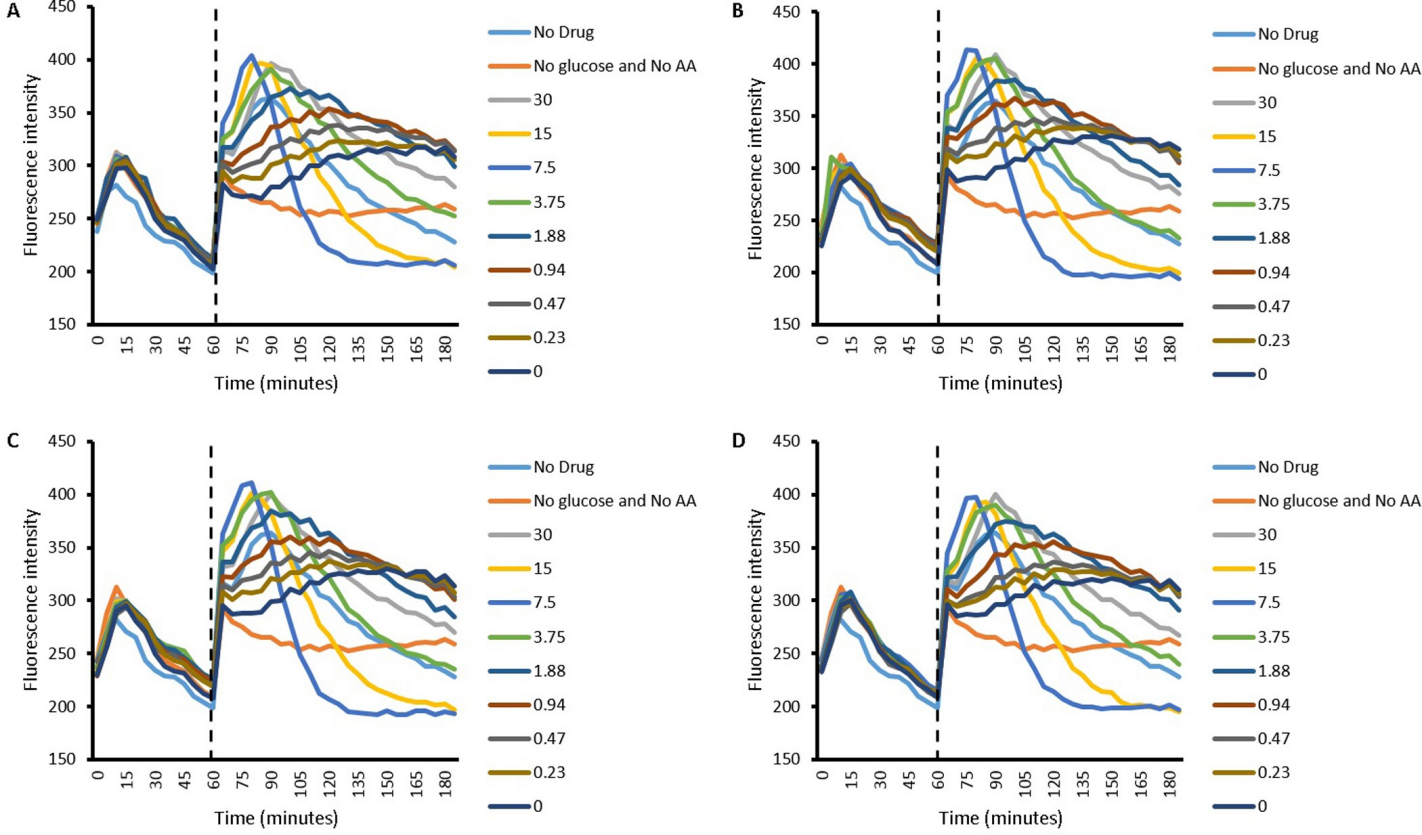
Efflux of EtBr by *S*. *aureus* in the presence of different concentrations of L-Asp (30 mM to 0.23 mM). A) 8.49 μM Cip B) 4.24 μM Cip C) 2.12 μM Cip D) 1.06 μM Cip. In the legend, numbers 0 to 30 represent amino acid concentrations in combination with respective Cip concentration. Firstly the dashed line represents end of first part of experiment where Cip and EtBr was allowed to accumulate within energy deprived cells and secondly the start of accumulation in the presence of the amino acid, with or without energy; n = 4.

**Fig 2:**
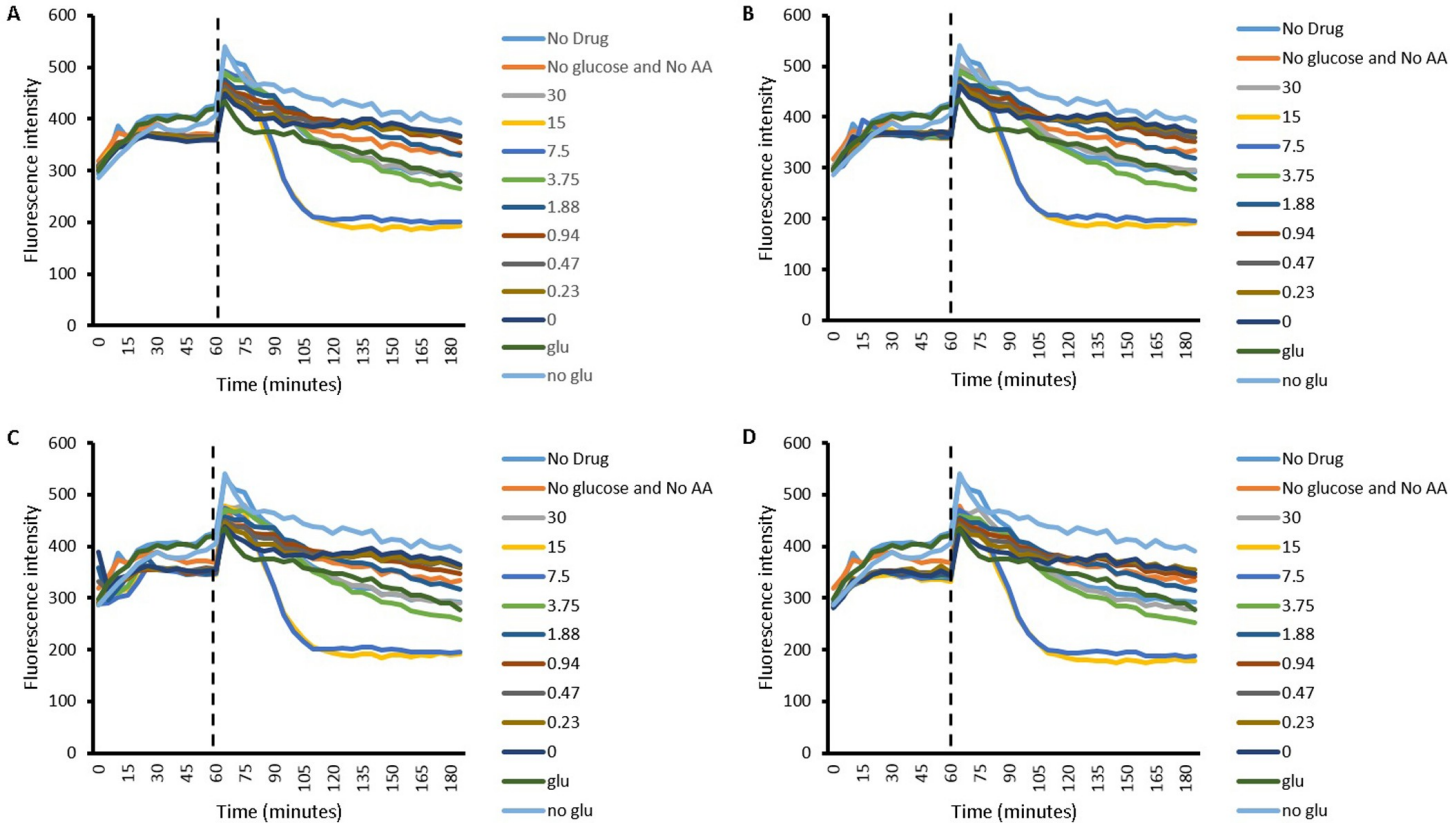
Efflux of EtBr by *S*. *aureus* in the presence of different concentrations of L-Glu (30 mM to 0.23 mM). A) 8.49 μM Cip B) 4.24 μM Cip C) 2.12 μM Cip D) 1.06 μM Cip. In the legend, numbers 0 to 30 represent amino acid concentrations in combination with respective Cip concentration. Firstly, the dashed line represents end of the first part of experiment where Cip and EtBr was allowed to accumulate within energy deprived cells and secondly the start of accumulation in the presence of the amino acid, with or without energy; n = 4.

Figs 1 and 2 presents the accumulation of EtBr in the presence of Cip on its own and along with its combinations with different concentrations of L-Asp (Fig 1) and L-Glu (Fig 2) in *S*. *aureus*. A striking observation is that the pattern of accumulation for each concentration of Cip used is similar. In other words no difference in the pattern of accumulation was observed between Cip concentration of 8.49 μM, 4.24 μM, 2.12 μM and 1.06 μM. After 60 minutes, which represents the second phase of accumulation assessment in the accumulation experiment, a general rise in fluorescence intensity is observed. This indicates a rise in intracellular EtBr. After this rise, a fall in fluorescence intensity is observed, potentially indicating EtBr efflux. Generally, a concentration dependent pattern in the rise and fall of fluorescence intensity is observed between 7.5 mM and 0 mM of either amino acid; as the concentration increases the rise as well as the fall in fluorescence intensity is steeper, or it can be said that the rate of both influx and efflux of EtBr decreases with a decrease in concentration of the amino acid which is especially true for L-Asp. The main outlier in this regard is Cip in combination with 30 mM amino acid. In other words whilst amino acid concentrations of 15 mM and 7.5 mM resulted in faster efflux of EtBr from within the cells, the concentrations of 0 mM, 0.23 mM and 0.47 mM showed slowest efflux, only surpassed by the control (No glu) which lacked the energy/glucose required for efflux. Whilst lack of glucose (no glu) followed a similar pattern, least increase in fluorescence intensity was observed with the presence of glucose (glu); both of these controls lack both Cip and L-Glu, the only difference being the absence or presence of glucose, respectively. Interestingly, 15 mM and 7.5 mM do not follow the pattern of slow reduction in fluorescence intensity and instead both showed a steep reduction in fluorescence intensity which plateaus to a lower level then ever observed in part one or two of the experiment. Furthermore, the pattern of efflux observed for 15 mM and 7.5 mM L-Glu was similar to the pattern observed with L-Asp of the same concentrations, in the sense that these concentrations lead to the greatest rate of reduction in fluoresce intensity after the initial rise. Although the energised and the energy deprived controls ‘glu’ and ‘no glu’ were not included in the experiment with the amino acid L-Asp, it would be fair to assume that they follow a similar pattern as when efflux experiments on *S*. *aureus* with L-Glu were conducted; there is no variable that should cause a distinction between the two set of experiments.

#### 2.2.2 Evaluation of the effect of amino acids, Cip and their combinations on the growth of *S*. *aureus*

Although a synergy in the pattern of efflux of EtBr was observed with both L-Asp and L-Glu when they were combined with the drug, it raised questions with regards to whether or not this translated into enhanced effectiveness of Cip against *S*. *aureus*. To study this, growth curves were done to look at the effect of combining L-Asp or L-Glu with the drug on the growth of *S*. *aureus* over a period of 16 hours.

Both here, and in section 2.3.2 which presents growth curves of *P*. *aeruginosa*, the curves are presented alongside each other with the same axis, enabling easy comparison between curves. Whilst growth curves were done to determine whether enhanced antimicrobial activity could be achieved by combining amino acids and Cip, the growth of both *S*. *aureus* and *P*. *aeruginosa* were also studied in the presence of both amino acids on their own as well as Cip on its own. By having these controls, the effect of amino acid (L-Asp or L-Glu) on its own and Cip on its own can be compared to their combinations. In cases where combination showed greater reduction in growth of the bacteria compared to both the drug and the amino acid in isolation, enhanced antimicrobial activity was concluded.

**Fig 3:**
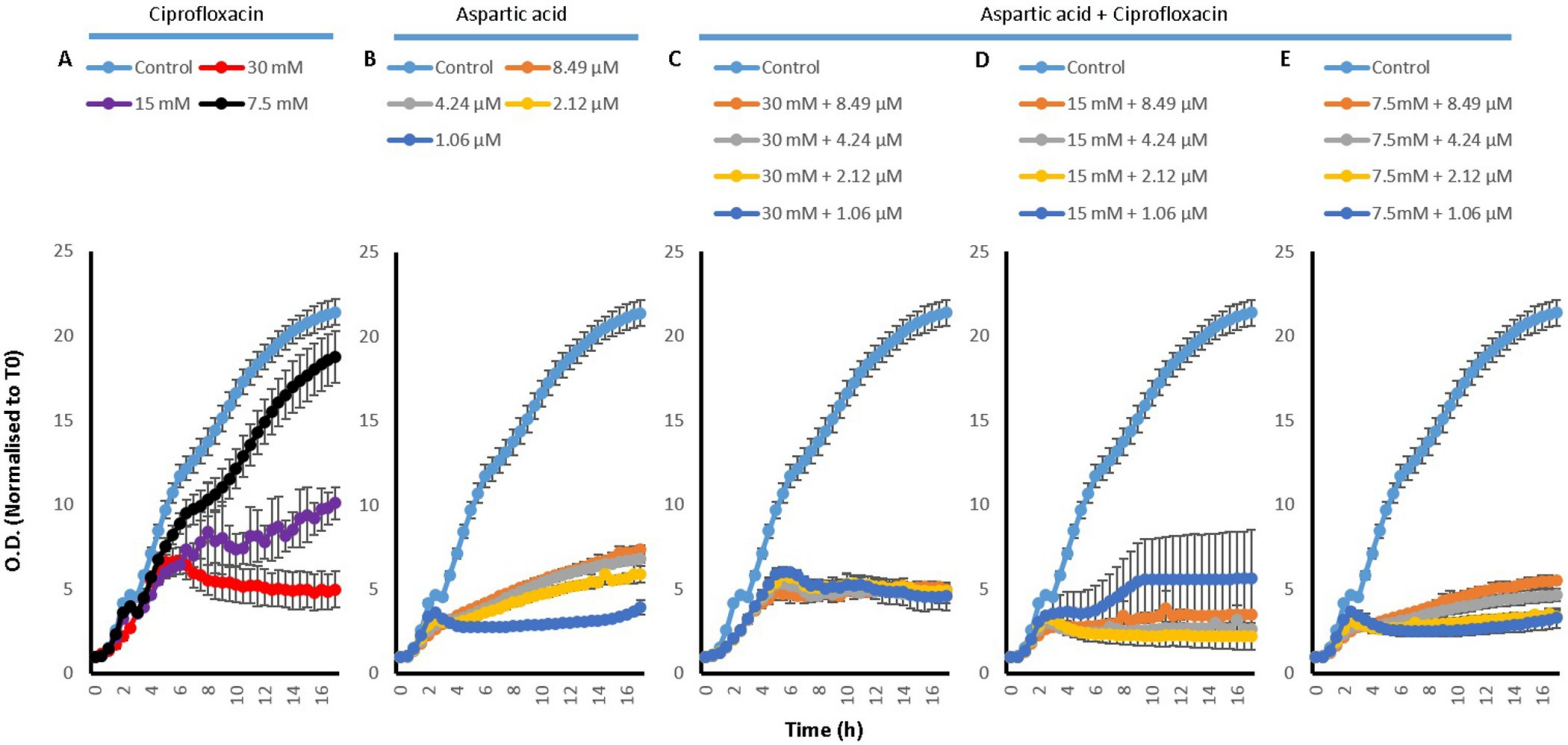
*S*. *aureus* growth curves with A) 30, 15 and 7.5 mM L-Asp amino acid on its own, B) with 8.49, 4.24, 2.12 and 1.06 μM Cip on its own, C) 30 mM L-Asp combined with 8.49, 4.24, 2.12 and 1.06 μM Cip, D) 15 mM L-Asp combined with 8.49, 4.24, 2.12 and 1.06 μM Cip and E) 7.5 mM L-Asp combined with 8.49, 4.24, 2.12 and 1.06 μM Cip; n = 3.

Growth curves of *S*. *aureus* in the study with L-Asp are presented in Fig 3. The curves include those of L-Asp (Fig 3A) and Cip (Fig 3B) both on their own and Cip concentrations of 8.49 μM, 4.24 μM, 2.12 μM and 1.06 μM in combination with L-Asp concentrations of 30 mM (Fig 3C), 15 mM (Fig 3D) and 7.5 mM (Fig 3E). A concentration dependent effect of L-Asp was observed on the growth of *S*. *aureus*; as the concentration of L-Asp is increased from 7.5 mM to 30 mM, a higher inhibitory effect on *S*. *aureus* growth is observed. Cip on its own also affected the growth of *S*. *aureus*. The concentration of L-Asp which seems to best enhanced Cip activity was 15 mM, since apart from its combination with 1.06 μM Cip, the combinations noticeably hindered the growth of *S*. *aureus* in comparison to the corresponding Cip concentrations on their own as well as 15 mM L-Asp on its own. Combinations between 7.5 mM L-Asp and Cip, again excluding 1.06 μM Cip, also showed some enhancement in antimicrobial activity. No difference was observed when Cip was combined with 30 mM L-Asp.

**Fig 4:**
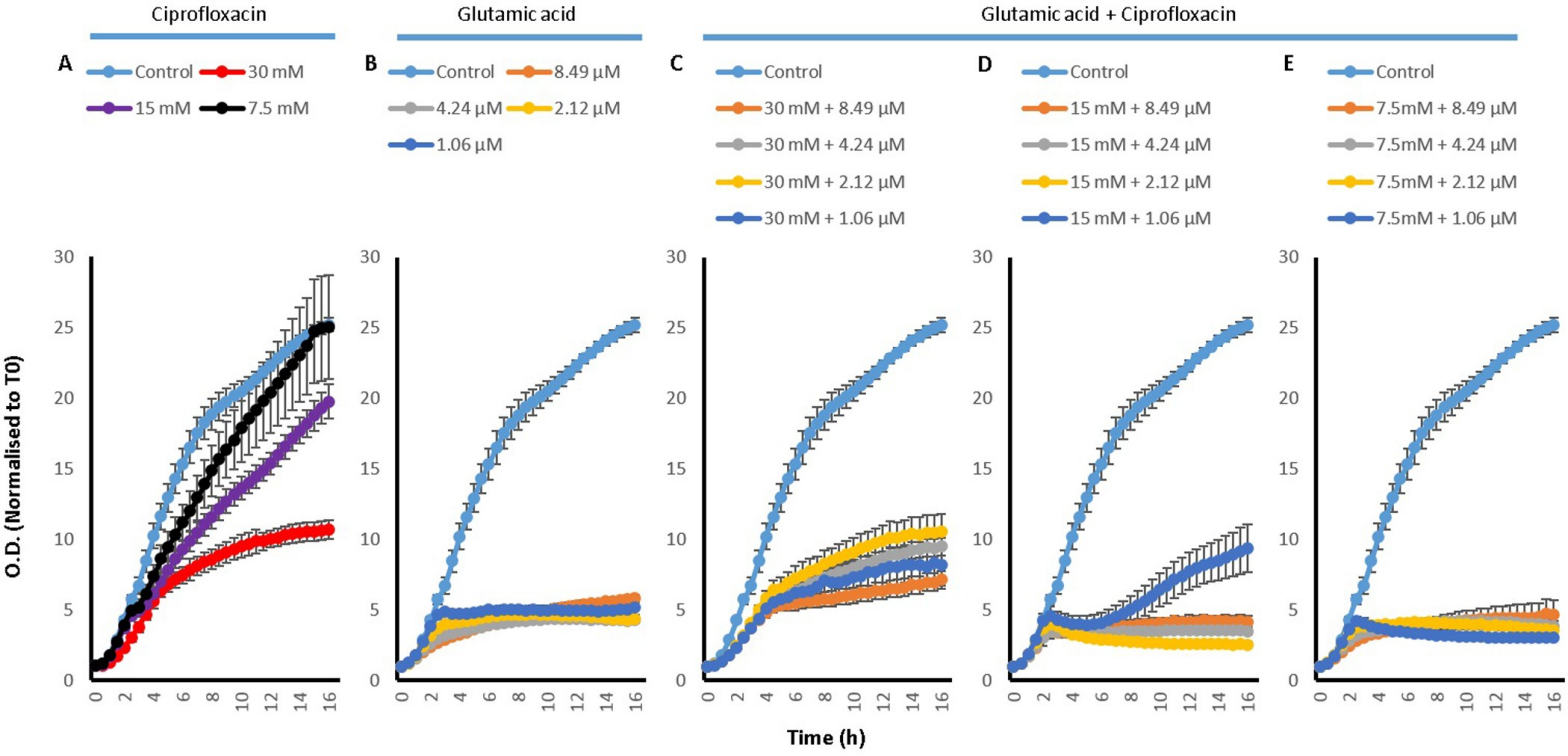
*S*. *aureus* growth curves with A) 30, 15 and 7.5 mM L-Glu amino acid on its own, B) with 8.49, 4.24, 2.12 and 1.06 μM Cip on its own, C) 30 mM L-Glu combined with 8.49, 4.24, 2.12 and 1.06 μM Cip, D) 15 mM L-Glu combined with 8.49, 4.24, 2.12 and 1.06 μM Cip and E) 7.5 mM L-Glu combined with 8.49, 4.24, 2.12 and 1.06 μM Cip; n = 3.

When investigating the growth of *S*. *aureus* in the presence of L-Glu (Fig 4A), Cip (Fig 4B), and L-Glu and Cip combinations (Fig 4C–4E), similar results to L-Asp were obtained. Both L-Glu and Cip on its own affected the growth of *S*. *aureus*. However no substantial difference was observed in the extent of growth inhibition between the different concentrations of Cip used. On the other hand, a concentration dependent inhibition in *S*. *aureus* growth was observed with L-Glu where apart from 7.5 mM, substantial inhibition was observed and increasing the concentration of the amino acid resulted in greater modulation of *S*. *aureus* growth. Like with L-Asp, combining Cip with15 mM and 7.5 mM L-Glu showed best enhancement in antimicrobial activity, where apart from combining with 1.06 μM L-Glu, all other combinations reduced *S*. *aureus* growth greater than their corresponding Cip and L-Glu concentrations. Cip combination with 7.5 mM L-Glu is noticeable because it not only modulated *S*. *aureus* growth to a similar level as 15 mM combinations does, the reduction was much greater for 7.5 mM combination than for 15 mM combination when compared to their corresponding amino acid concentration on its own; 7.5 mM L-Glu did not affect particularly *S*. *aureus* growth. Again no difference was observed when Cip was combined with 30 mM L-Glu compared to the corresponding concentrations on their own.

### 2.3 Efflux assessment in *P*. *aeruginosa*

Like with *S*. *aureus*, the efflux of EtBr in the presence of amino acids, Cip and their combinations was also investigated for *P*. *aeruginosa*. The concentrations of Cip used were 1.06 μM, 0.53 μM, 0.27 μM and 0.13 μM. The amino acids used to investigate this were again L-Asp and L-Glu. Each Cip concentration mentioned above was studied individually as well as in combination with L-Asp and L-Glu concentrations of 30 Mm,15 mM, 7.5 mM, 3.75 mM, 1.88 mM, 0.94 mM, 0.47 mM and 0.23 mM.

#### 2.3.1 Efflux of EtBr in *P*. *aeruginosa*

**Fig 5:**
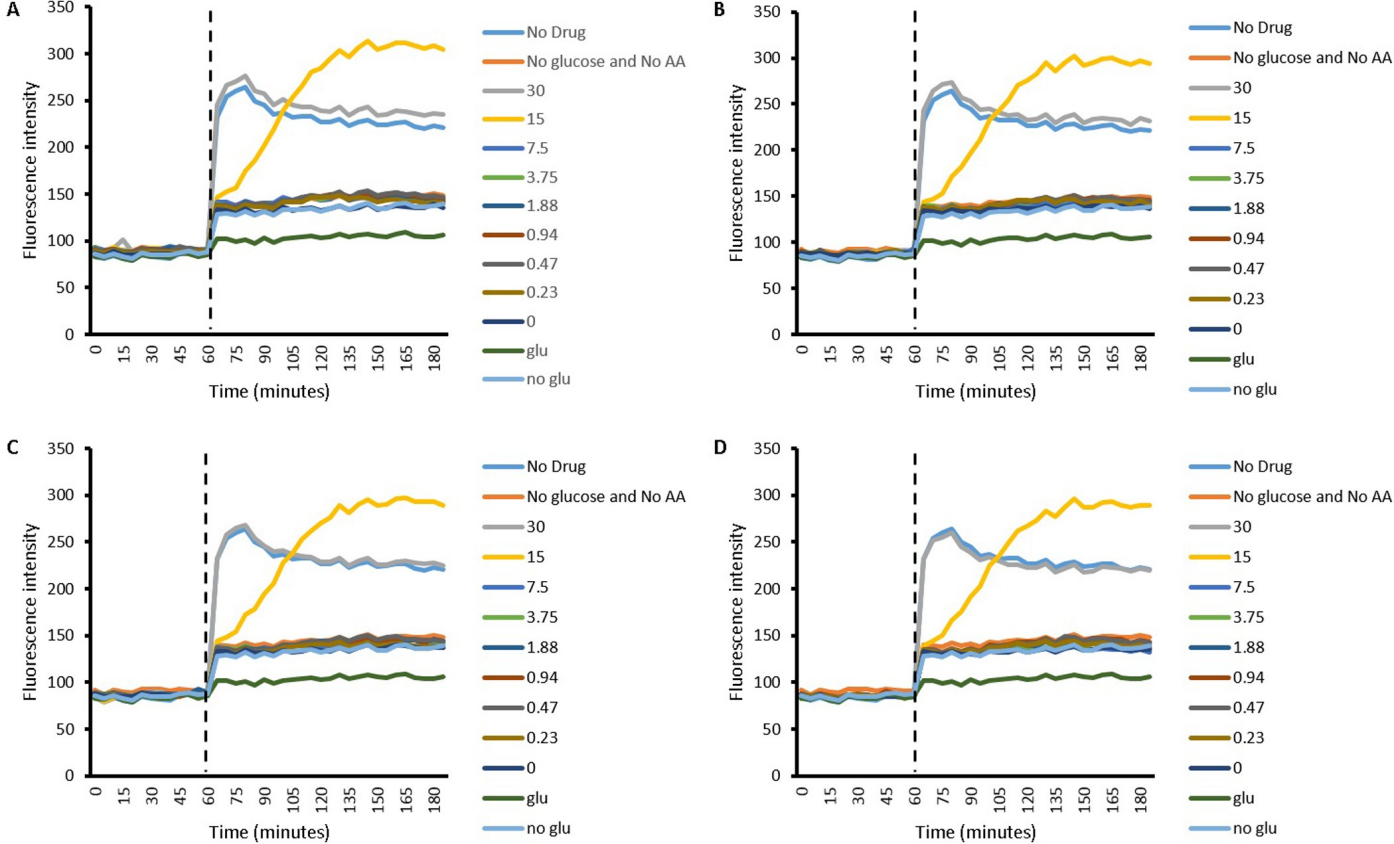
Efflux of EtBr by *P*. *aeruginosa* in the presence of different concentrations of L-Asp (30 mM to 0.23 mM). A) 1.06 μM Cip B) 0.53 μM Cip C) 0.27 μM Cip D) 0.13 μM Cip. In the legend, numbers 0 to 30 represent amino acid concentrations in combination with respective Cip concentration. Firstly, the dashed line represents end of the first part of experiment where Cip and EtBr was allowed to accumulate within energy deprived cells and secondly the start of accumulation in the presence of the amino acid, with or without energy; n = 4.

**Fig 6:**
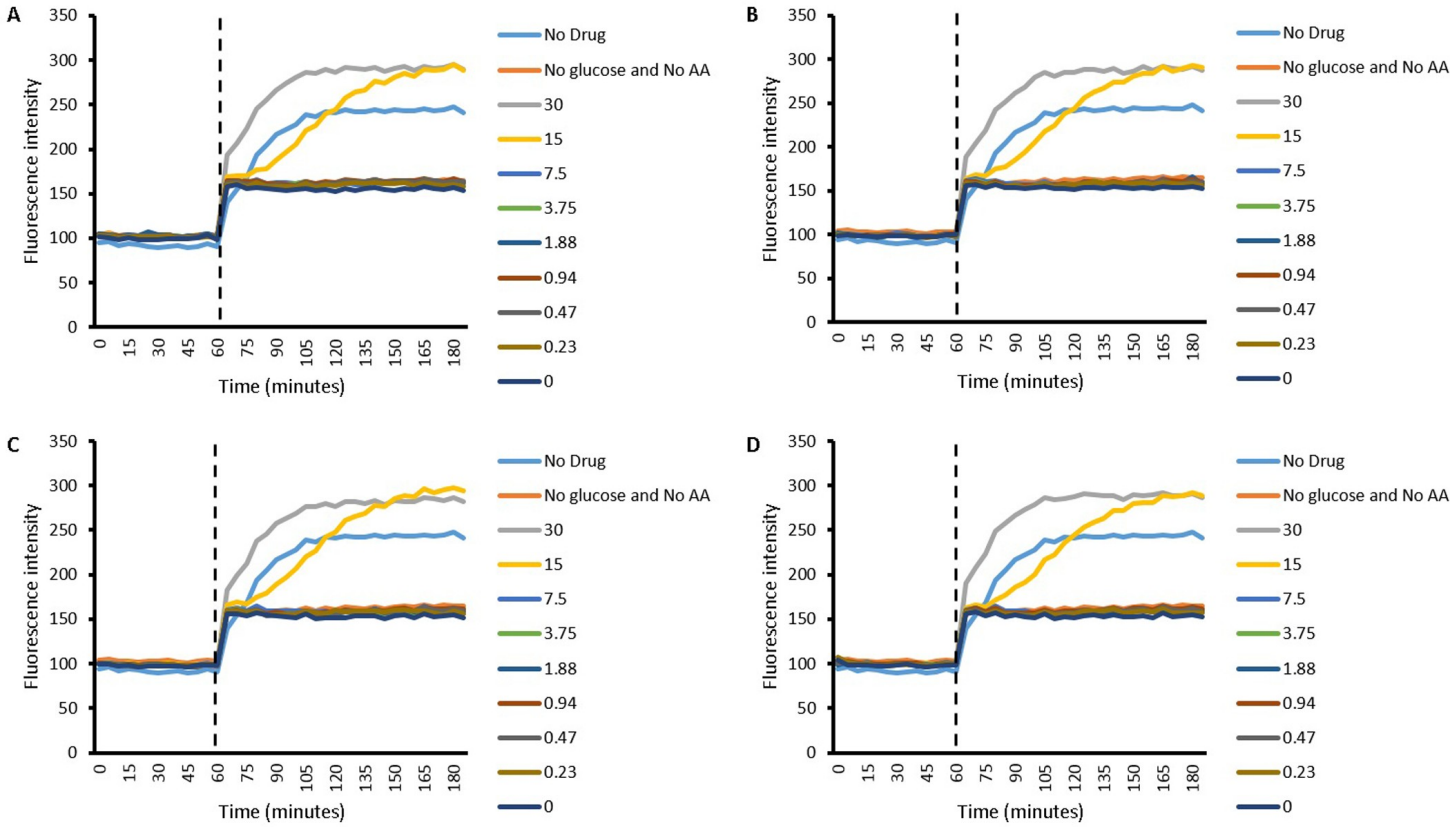
Efflux of EtBr by *P*. *aeruginosa* in the presence of different concentrations of L-Glu (30 mM to 0.23 mM). A) 1.06 μM Cip B) 0.53 μM Cip C) 0.27 μM Cip D) 0.13 μM Cip. In the legend, numbers 0 to 30 represent amino acid concentrations in combination with respective Cip concentration.Firstly, the dashed line represents end of the first part of experiment where Cip and EtBr was allowed to accumulate within energy deprived cells and secondly the start of accumulation in the presence of the amino acid, with or without energy; n = 4.

Efflux of EtBr in *P*. *aeruginosa* was also investigated in the presence of Cip, amino acids and with Cip in combination with different concentrations of L-Asp and L-Glu and is presented in Figs 5 and 6, respectively. As with *S*. *aureus*, the pattern of efflux of EtBr obtained with all of the Cip concentrations used was similar with no observable difference. Furthermore, the pattern of efflux was similar irrespective of the amino acids used. For both amino acids, in the second part of the accumulation experiment, a general rise in fluorescence intensity is observed, indicating a rise in intracellular accumulation of EtBr. The patten of efflux is startlingly different to that observed with *S*. *aureus*; a very distinct pattern of efflux was observed with *P*. *aeruginosa*. Glucose caused a minimal rise in fluorescence intensity and Cip combination with 30 mM and 15 mM L-Asp as well as ‘no drug’ which also contains 30 mM L-Asp, caused the largest increase in fluorescence intensity. The rest of the combinations, along with the energy deprived ‘no glu’ control present a moderate rise in fluorescence above ‘glu’. This is true for both of the amino acids used. The only distinction between the two is that with L-Asp, the curve for 30 mM L-Asp whether with Cip or without (no drug) showed a decrease in fluorescence intensity following the initial rise, indicating a decrease in intracellular EtBr. Although the energised and the energy deprived controls ‘glu’ and ‘no glu’ were not included in the experiment with the amino acid L-Glu, it is expected they followed a similar pattern as when efflux experiments on *P*. *aeruginosa* with L-Asp were conducted.

#### 2.3.2 Evaluation of the effect of amino acids, Cip and their combinations on the growth of *P*. *aeruginosa*

To investigate how the effect of amino acids, Cip and their combinations on the efflux of EtBr translated into the efficacy of Cip on the bacteria, growth curves were also conducted for *P*. *aeruginosa*. For this an untreated control was used along with the treatments of Cip, amino acids and combinations of the two.

**Fig 7:**
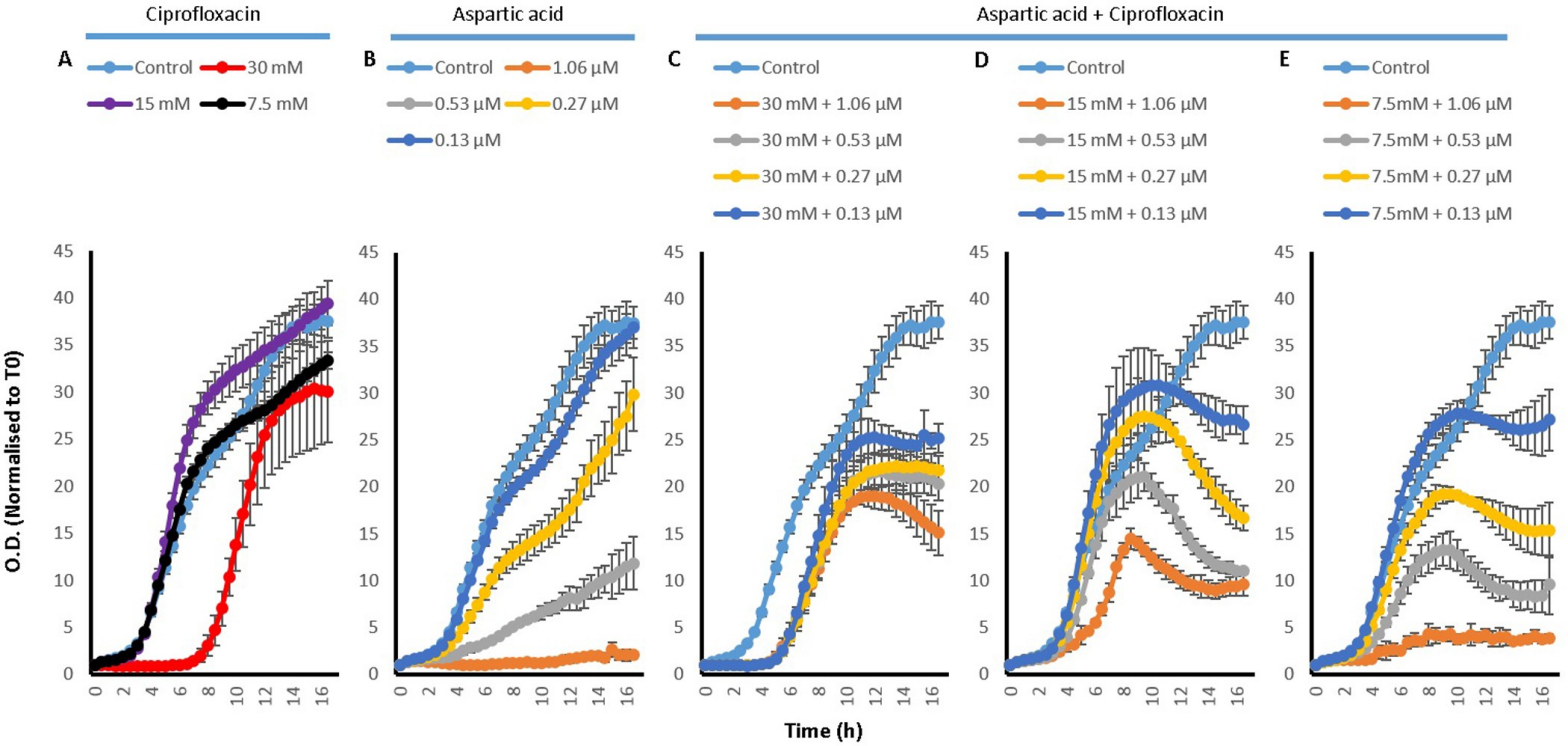
*P*. *aeruginosa* growth curves with A) 30, 15 and 7.5 mM L-Asp amino acid on its own, B) with 1.06, 0.53, 0.27 and 0.13 μM Cip on its own, C) 30 mM L-Asp combined with 1.06, 0.53, 0.27 and 0.13 μM Cip, D) 15 mM L-Asp combined with 1.06, 0.53, 0.27 and 0.13 μM Cip and E) 7.5 mM L-Asp combined with 1.06, 0.53, 0.27 and 0.13 μM Cip; n = 3.

**Fig 8:**
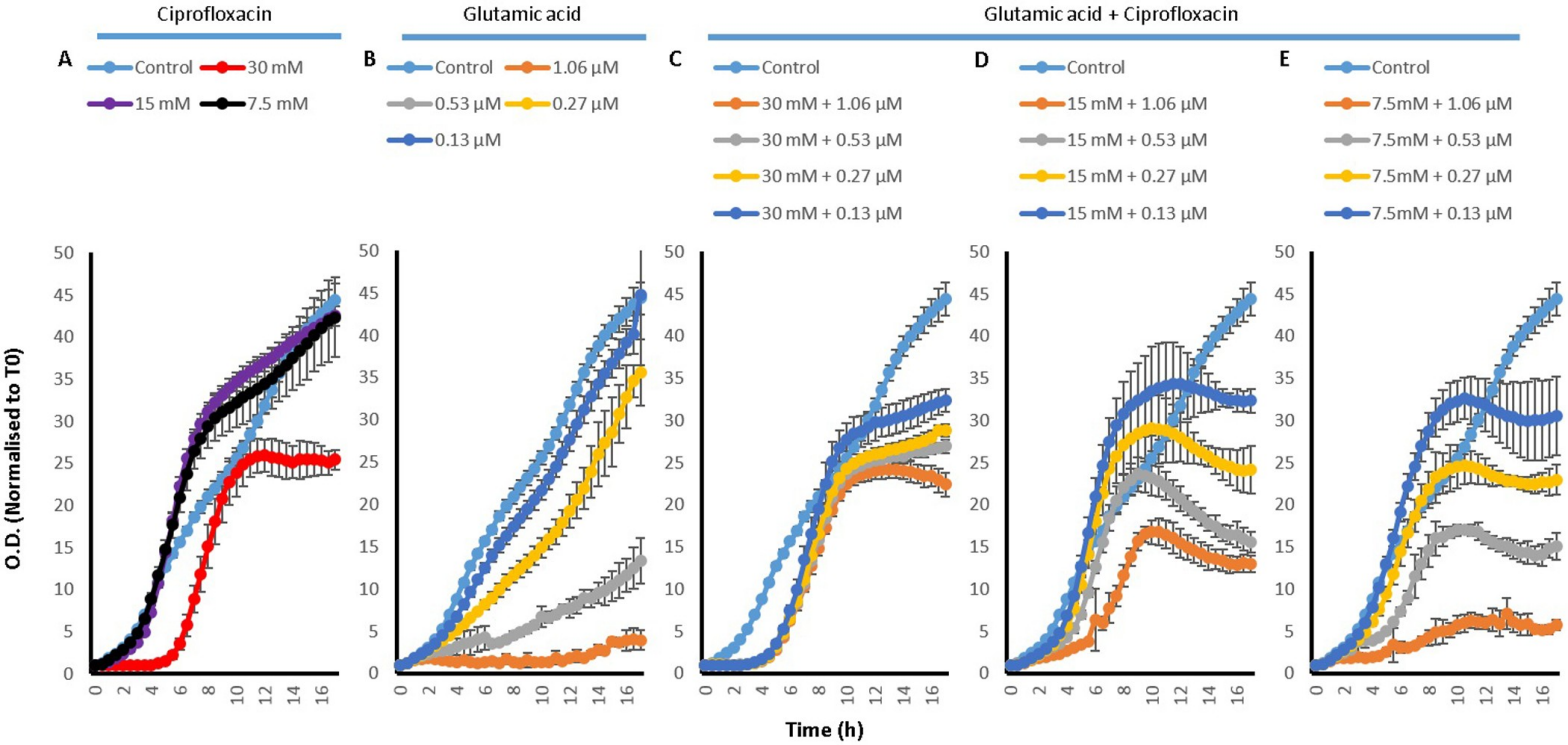
*P*. *aeruginosa* growth curves with A) 30, 15 and 7.5 mM L-Glu amino acid on its own, B) with 1.06, 0.53, 0.27 and 0.13 μM Cip on its own, C) 30 mM L-Glu combined with 1.06, 0.53, 0.27 and 0.13 μM Cip, D) 15 mM L-Glu combined with 1.06, 0.53, 0.27 and 0.13 μM Cip and E) 7.5 mM L-Glu combined with 1.06, 0.53, 0.27 and 0.13 μM Cip; n = 3.

Growth curves of *P*. *aeruginosa* treated with L-Asp (Fig 7) and L-Glu (Fig 8) and their combinations with Cip showed very similar results. Whilst Cip has a concentration dependent effect on the growth of *P*. *aeruginosa* (Figs 7 and 8), where a higher concentration hindered more greatly the growth of the bacteria, L-Asp on its own was generally unable to affect the growth of *P*. *aeruginosa*; only a delay in the onset of the exponential phase was observed with 30 mM L-Asp. The only concentration of L-Glu which somewhat inhibited *P*. *aeruginosa* growth is 30 mM L-Glu. Although, combining Cip with L-Asp lead to an early onset death phase, a very interesting pattern was observed. It seemed that when an amino acid was combined with higher concentration of Cip, it reduced the effectiveness of Cip and enhanced *P*. *aeruginosa* growth, whereas if the amino acid was combined with lower concentrations of Cip, it resulted in an enhanced effectiveness of Cip with a further inhibition of the growth of *P*. *aeruginosa*. For example, combining 30, 15 or 7.5 mM of L-Asp with Cip concentrations 1.06 and 0.53 μM seemed to aid the growth of *P*. *aeruginosa*, whilst combining with 0.27 and 0.13 μM does seem result in increased antimicrobial activity as see by an the early onset of death phase mentioned earlier. Thus, enhancement in the antimicrobial activity of Cip was observed in two main ways. Firstly combining the drug with the amino acids results in an earlier onset of the death phase. And secondly, combining 15 and 7.5 mM of the amino acids with 0.27 and 0.13 μM Cip leads to less overall *P*. *aeruginosa* growth. For the duration of the experiment, the least increase in the activity of Cip was observed with combinations which contained 30 mM of the amino acids.

#### 2.3.3 Evaluation of the effect of ciprofloxacin and amino acids on pigment production

**Fig 9:**
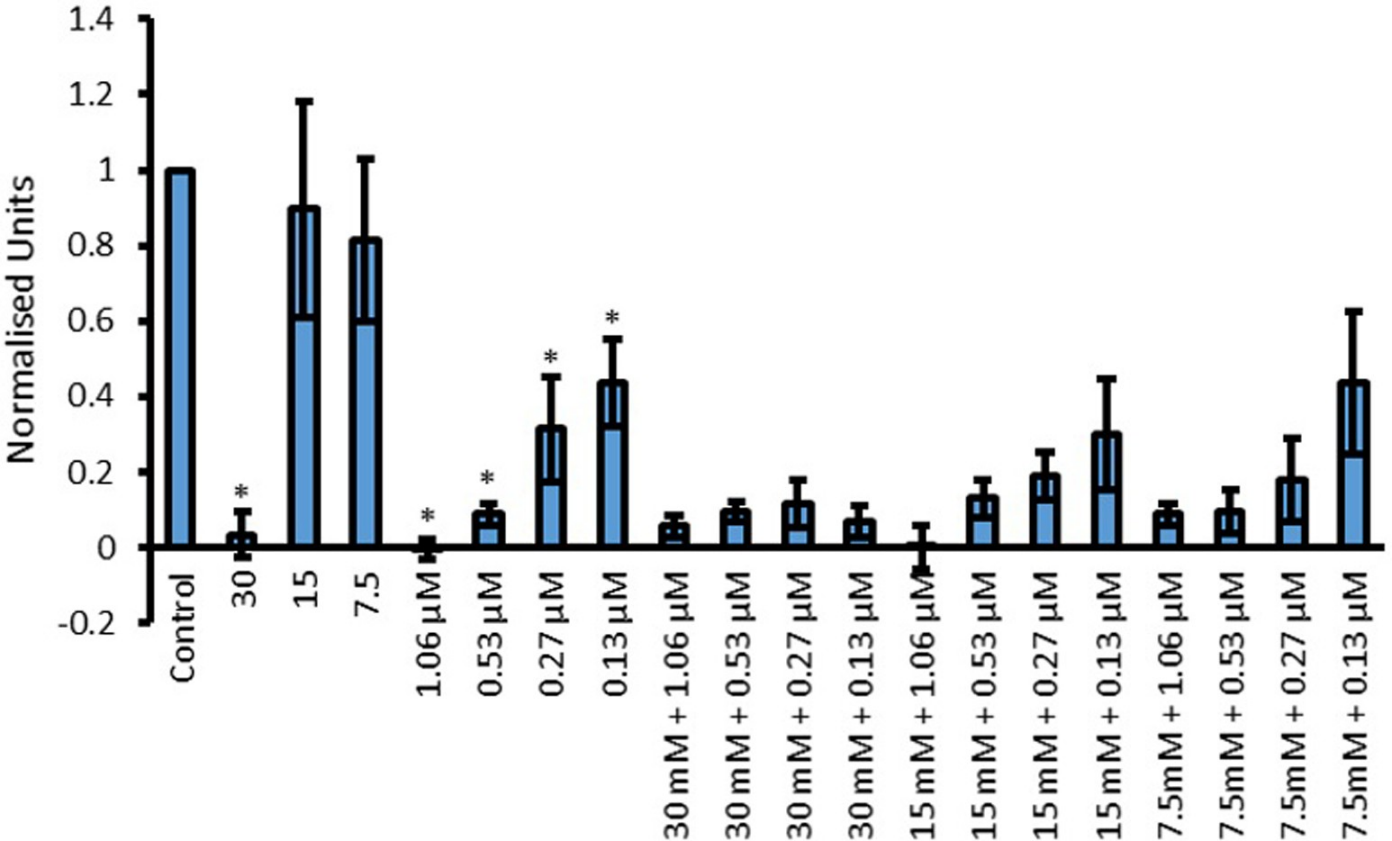
Effect of L-Asp, Cip and L-Asp with Cip combinations on the production of pyocyanin by *P*. *aeruginosa*. Y-axis shows concentration of pyocyanin in normalized units when treated with 30, 15 and 7.5 mM L-Asp, 1.06, 0.53, 0.27 and 0.13 μM Cip and their combinations represented on the X-axis. Significant results are indicated by *, where * represent a significant reduction in pigment production by the amino acid or Cip compared to control (p < 0.05 was taken as significant); n = 3.

**Fig 10:**
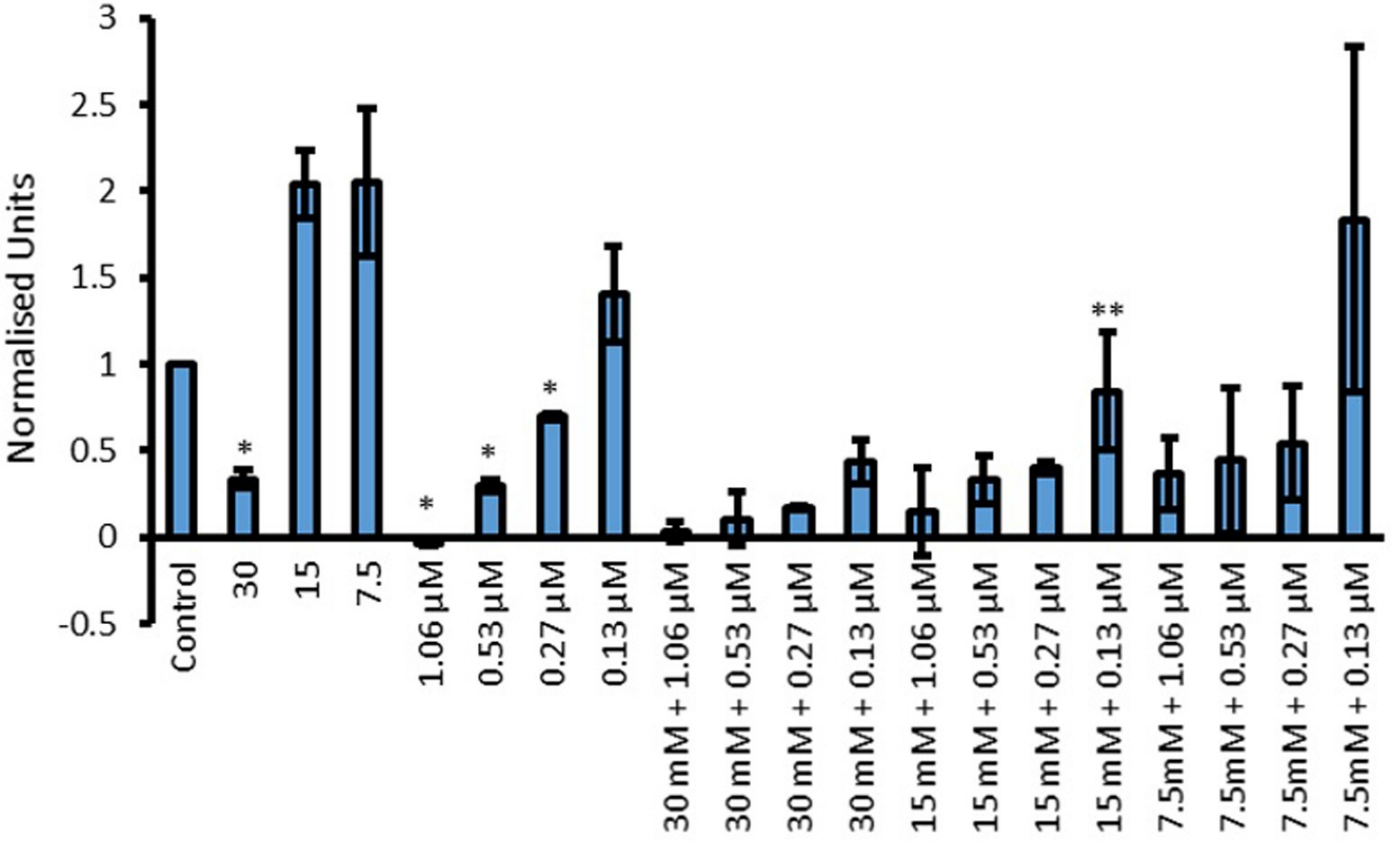
Effect of L-Glu, Cip and L-Glu with Cip combinations on the production of pyocyanin by *P*. *aeruginosa*. Y-axis shows concentration of pyocyanin in normalized units when treated with 30, 15 and 7.5 mM L-Glu, 1.06, 0.53, 0.27 and 0.13 μM Cip and their combinations. Significant results are indicated by * or **, where * represent a significant reduction in pigment production by the amino acid or Cip compared to control and ** represents a significant reduction in pigment production by combinations (AA + Cip) compared to both of the individual corresponding components (p < 0.05 was taken as significant); n = 3.

Pigment production during the growth curve experiments varied depending on the combinations of Cip and amino acids that *P*. *aeruginosa* was exposed to. Due to this, it was appropriate to quantify the pigments produced. Pyocyanin production and was quantified using the chloroform/HCl extraction method. A relation between the pigment intensity was observed where a more intense green pigmentation corresponded to higher amounts of pyocyanin (Figs 9B and 10B) present. For statistical analysis, initially one way ANOVA was conducted to see whether there was significant difference within the data or not. This was followed by the uncorrected Fisher’s LSD test to see where this significant difference lies. Fisher’s LSD test was chosen over tests which correct for multiple comparisons because in answering the hypothesis, each comparison stood alone. In other words, each specific combination of amino acid and the drug was compared to both of its components in isolation. It was not relevant to compare each specific combination with other concentrations.

Fig 9 presents pyocyanin production by *P*. *aeruginosa* when incubated with L-Asp, Cip and various L-Asp and Cip combinations. Cip showed a statistically significant (p<0.0001) concentration dependent reduction in pyocyanin production where a higher concentration of the drug led to less pigment production. The only L-Asp concentration which resulted in significant pigment reduction was 30 mM (p<0.0001). When combining Cip and L-Asp, enhanced antimicrobial activity was confirmed if a particular combination resulted in less pigment production compared to both of its corresponding parts. If combination only resulted in less pigment production compared to one of the corresponding part, no further enhancement in the antimicrobial activity was confirmed. Although all combinations significantly (p<0.0001) reduced the amount of pyocyanin produced by *P*. *aeruginosa*, none seem to show statistically significant reduction in pigment production compared to both of its parts in isolation. Using 0.27 and 0.13 μM Cip combined with 30 mM L-Asp showed a significant reduction compared to the corresponding Cip concentrations, this was not significantly lower than 30 mM L-Asp on its own. It may be worth pointing out that although not statistically significant combinations, some further enhancement in antimicrobial activity was observed with the combinations of 15 mM + 0.27 μM, 15 Mm + 0.13 μM and 7.5 Mm + 0.27 μM.

Pyocyanin production by *P*. *aeruginosa* once treated with Cip, L-Glu and their combinations at various concentrations is presented in Fig 10. Interestingly, 15 and 7.5 mM L-Glu led to increased pyocyanin production whilst 30 mM significantly (p = 0.011) reduced its production. Cip concentrations of 1.06 (p<0.001) and 0.53 (p = 0.0078) reduced pyocyanin production in a concentration dependent manner. 0.13 μM Cip had no significant effect on pyocyanin production and although some reduction was observed with 0.27 μM, this was not statistically significant. As before, improved efficacy of Cip was established if combinations led to greater reduction in pyocyanin production compared to both Cip and L-Glu of the same concentrations in isolation. With this rule in mind, the only combination which showed substantial enhancement in antimicrobial activity was 0.13 μM Cip combined with 15 mM L-Glu (p = 0.031). Though Cip concentrations of 0.27 and 0.13 μM led to reduced pyocyanin production compared to the corresponding Cip concentrations, the amount of pyocyanin was not less than that produced by 30 mM L-Glu on its own. Hence these combinations were not taken to have improved Cip efficacy in preventing pyocyanin production.

**Fig 11:**
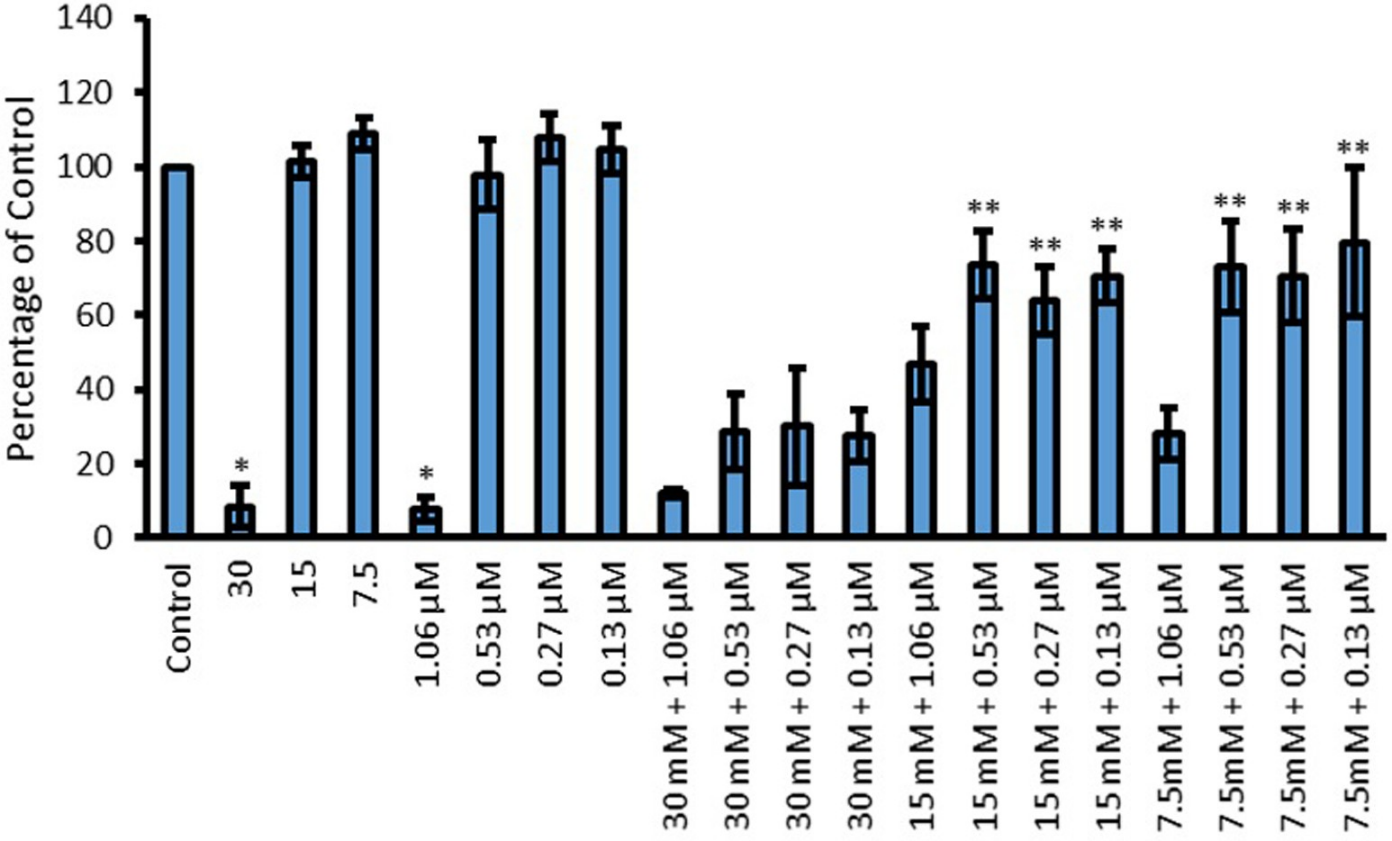
Effect of L-Asp, Cip and L-Asp and Cip combinations on the production of pyoverdine by *P*. *aeruginosa*. Y-axis shows concentration of pyoverdine as a percentage of untreated, when treated with 30, 15 and 7.5 mM L-Asp, 1.06, 0.53, 0.27 and 0.13 μM Cip and their combinations. Significant results are indicated by * or **, where * represent a significant reduction in pigment production by the amino acid or Cip compared to control and ** represents a significant reduction in pigment production by combinations (AA + Cip) compared to both of the individual corresponding components (p < 0.05 was taken as significant); n = 3.

Pyoverdine production by *P*. *aeruginosa* was also quantified. Its production when exposed to Cip, L-Asp and their combinations is presented in Fig 11. When used in isolation only 30 mM L-Asp (p<0.0001) and 1.06 μM Cip (p<0.0001) resulted in a significant reduction in pyoverdine production. Once Cip and L-Asp were combined, further reduction in pyoverdine production was observed using 15 mM (p<0.01) and 7.5 mM L-Asp (p<0.01) in combination with 0.53, 0.27 and 0.13 μM Cip. Although combining these Cip concentrations with 30 mM L-Asp hindered pigment production compared to the corresponding Cip concentrations, the amount produced was not lower than that produced with exposure to the corresponding amino acid treatment on its own. Likewise, even though combining 1.06 μM Cip with 15 and 7.5 mM L-Asp resulted in lower pyoverdine production compared to when these amino acid concentrations were used in isolation, the values obtained were not lower than that those obtained with 1.06 μM Cip. Hence no enhancement in antimicrobial activity can be attributed to these specific combinations.

**Fig 12:**
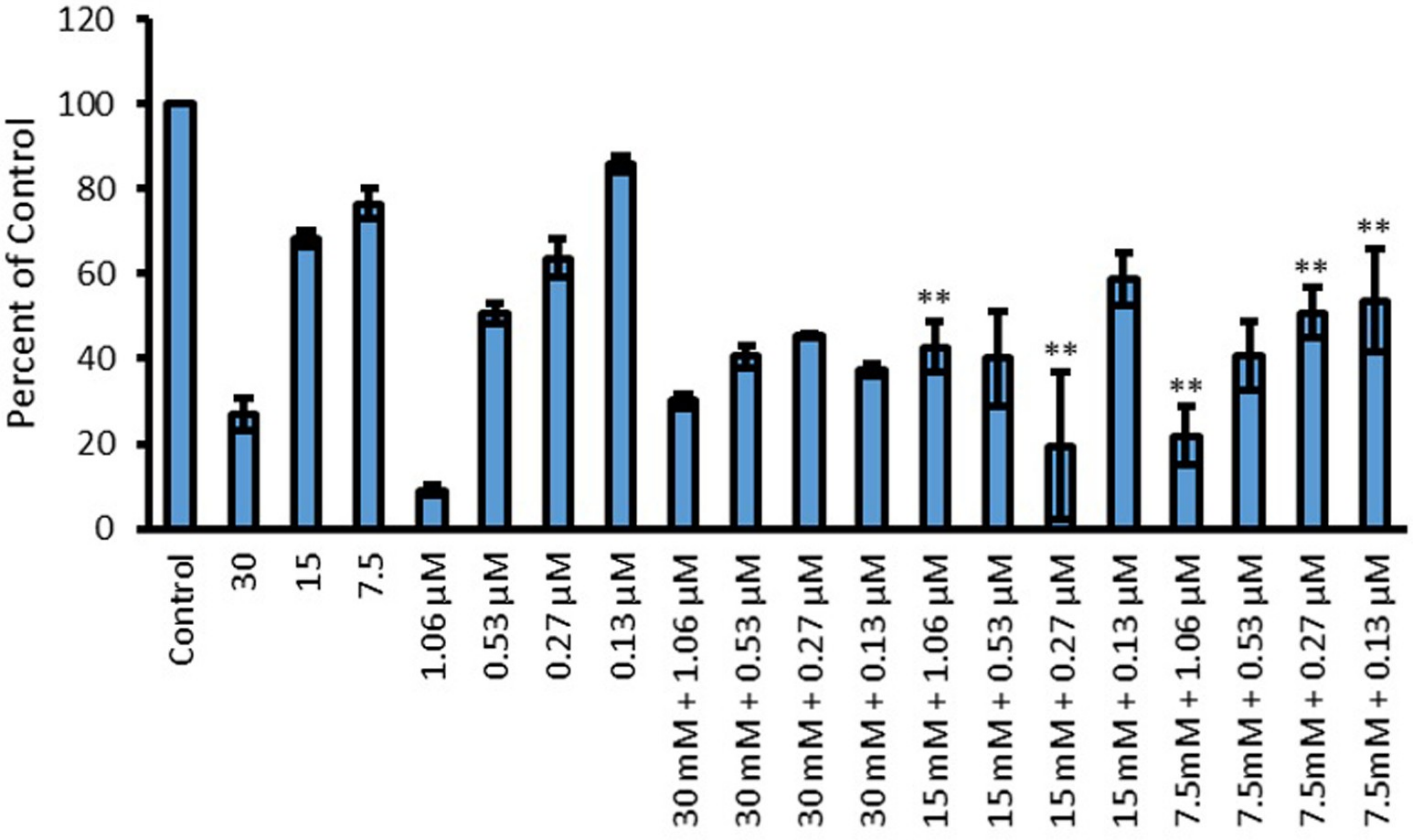
Effect of L-Glu, Cip and L-Glu and Cip combinations on the production of pyoverdine by *P*. *aeruginosa*. Y-axis shows concentration of pyoverdine as a percentage of untreated, when treated with 30, 15 and 7.5 mM L-Glu, 1.06, 0.53, 0.27 and 0.13 μM Cip and their combinations. Significant results are indicated by * or **, where * represent a significant reduction in pigment production by the amino acid or Cip compared to control and ** represents a significant reduction in pigment production by combinations (AA + Cip) compared to both of the individual corresponding components (p < 0.05 was taken as significant); n = 3.

Results for pyoverdine production when L-Glu was used as an accumulation enhancer for Cip are presented in Fig 12. Both L-Glu (p<0.001) and Cip (p<0.05) showed a concentration dependent reduction in pyoverdine production where a higher concentration of the amino acid and the drug resulted in less production of the pigment. Like with L-Asp, Cip combinations with 30 mM L-Glu did not result in any further enhancement of antimicrobial activity of Cip. This is because the combinations did not decrease pyoverdine production lower than the amount produced by 30 mM L-Glu on its own. The combinations which showed positive enhancement in antimicrobial activity, due to being more effective in inhibiting pigment production as compared to both of the corresponding parts, were 15 mM L-Glu combined with 1.06 (p<0.0001) and 0.27 μM (p<0.0001) Cip and 7.5 mM L-Glu combined with 1.06 (p = 0.022), 0.27 (p = 0.022) and 0.13 μM (p<0.0001) Cip.

## 3. Discussion

In a bid to overcome the ever growing resistance to antibiotics in clinical use, adjuvants are employed and also form part of the research area in the field of AMR. Generally, adjuvants aim to inhibit the resistance mechanism to particular drugs. For example, if an enzyme inactivates a drug, the adjuvant would act to increase the activity of the drug by inhibiting the enzyme. Likewise, if a drug is extruded out of efflux pumps, the adjuvant would act to inhibit this efflux [19–23].

Bacteria could still acquire resistance to adjuvants such as amino acids as demonstrated by the sulbactam-ampicillin example. The β-lactamase inhibitor sulbactam functions by inhibiting the β-lactamase enzyme that would otherwise inhibit the β-lactam class of antibiotics such as ampicillin [24]. Although sulbactam was approved for clinical use in 1986, persistence despite sulbactam-penicillin co-administration was reported as early as 1988 [25,26]. In United States, rate of resistance to sulbactam-ampicillin was reported to be 35.2% in the period of 2003 to 2005 and rose to 44.9% and 41.2% by the period of 2006 to 2008 and the period of 2009 to 2012 respectively [24,27]. Although substances capable of restoring sulbactam activity have been identified, this approach does not seem ideal; inhibiting the resistance to adjuvants which are meant to be inhibiting the resistance to antibiotics seems inefficient [28]. Furthermore, this may only be a temporary solution and resistance to such mechanisms will probably immerge.

Although sulbactam can confer intrinsic antibiotic activity, resistance is also likely to arise against adjuvants which do not [25]. This is because even with the lack of intrinsic activity, the indirect antibacterial activity exhibited by combinations of the adjuvants with an antibiotic, is likely to select for phenotypes resistant to the adjuvant.

Other forms of adjuvants are those which act by the inhibition of efflux pumps and modulation of membrane permeability. To enhance the accumulation of drugs in resistant bacteria, these are the two main strategies employed [19]. To our knowledge, naturally occurring acidic amino acids L-Asp and L-Glu have not been studied for their potential role as adjuvants which work by inhibiting efflux. Thus in this study, the amino acids which have already been proven to enhance the permeability of Cip as well as other drugs in Caco-2 mono layers, were investigated as potential adjuvants to Cip in enhancing the drugs antimicrobial activity in bacteria [3,29].

The results in this paper present that the accumulation of EtBr can be enhanced within bacteria with the use of acidic amino acids as counterions. This observation finds evidence in the efflux experiments, where the addition of both amino acids (after 60 min) causes intracellular levels of EtBr within *S*. *aureus* and *P*. *aeruginosa* to rise (Figs 1, 2, 5 and 6). Since Cip is expected to compete with EtBr for efflux, the EtBr efflux assays carried out in the presence of Cip may give indications of the drugs accumulation or efflux behavior. It is worth highlighting however, that the current work does not give evidence to this, and further experimentation, such as those measuring accumulation of Cip directly would be needed to confirm this. Further experimentation may also prove the method used here, once thoroughly validated used here to be a novel in indirectly determining efflux behavior of drugs.

Nevertheless, the increase in accumulation of EtBr in the presence or absence of Cip in both bacteria gives evidence to support that amino acids are able to modulate efflux or transport mechanism of bacteria. Thus it can be concluded that amino acids may also be able to effect the accumulation or efflux of other substrates of efflux too, such as Cip. Although this is not yet concluded from the presented data, it would not be appropriate to assume that similar efflux modulating mechanisms are involved in enhancing the antimicrobial activity observed with the growth curves and with pigment production analysis.

With *S*. *aureus* the effect on EtBr accumulation is amino acid concentration dependent. On the other hand, though with *P*. *aeruginosa*, enhancement in accumulation only occurs with the two highest amino acid concentrations used, the efflux pattern when 15 and 30 mM L-Asp or L-Glu is used, is strikingly different to that observed with *S*. *aureus*. This in turn presents as further reduction in *S*. *aureus* and *P*. *aeruginosa* growth. Interestingly, whilst studying growth of bacteria, a reduction in toxic pigment production by *P*. *aeruginosa* was also observed and later quantified. Both pyocyanin and pyoverdine have been linked to the generation of reactive oxygen species, rendering the pigments cytotoxic in nature and making them clinically relevant. Furthermore, acting as a siderophore, pyoverdine also enables *P*. *aeruginosa* to acquire more iron needed for its metabolic functioning [30]. Pyocyanin is also knows to play substantial role in the formation of *P*. *aeruginosa* biofilms. It enhances eDNA release whilst also binding to it and thus enhancing bacterial aggregation [31,32]. Thus, although reduction in pyocyanin production may be a result of reduced bacterial growth, nevertheless in addition to the hypothesis presented by our group previously, the ultimate reduction in the pigment may be another way acidic amino acids exhibit anti-biofilm activity [2].

The findings presented in this paper are similar to work done with another class of antibiotic by our group, tetracycline (S1–S12 Figs). This work also showed that L-Asp and L-Glu are able to enhance EtBr accumulation within both *S*. *aureus* (S1 and S2 Figs) and *P*. *aeruginosa* (S5 and S6 Figs), even in the presence of TC. Work with TC confirmed that amino acids, when combined with a drug which in this case was TC, are able to exhibit enhanced antimicrobial activity. Again, this was evident from the inhibitory effects on the growth of both bacteria (S3, S4, S7 and S8 Figs) as well as reduced pigment production (S9–S12 Figs) by *P*. *aeruginosa*. This shows that L-Asp and L-Glu exhibit their effect through a mechanism which is independent to the drug used and thus further hints towards a universal mechanism such as drug permeation or efflux being modulated.

In this regards, it is useful to consider here that TC acts on the 30S ribosomal subunit to inhibit protein synthesis whilst Cip has its inhibitory affect by acting on DNA-gyrase and DNA topoisomerase IV, inhibiting cell replication. Meaning, the two mechanisms of action are completely different. Due to this it is likely that the amino acids work in a manner which is independent to interfering with these mechanisms. Substantiating the possibility that amino acids act by a mechanism which interferes with a mutually shared process subjected to both antibiotics such as those mentioned earlier. Some of these mechanisms will be considered later on.

It is possible that the differences in the efflux patterns of EtBr observed with *S*. *aureus* and *P*. *aeruginosa* are due to the differences in the membranes of the respective bacteria. Being Gram-positive *S*. *aureus* has only one membrane whilst the Gram-negative *P*. *aeruginosa* has an outer and inner membrane. As mentioned earlier the outer membrane of Gram-negative bacteria confers substantial resistance to the uptake of antibiotics. One mechanism which may underpinning the enhanced antimicrobial activity could be enhanced drug uptake. If this is the case, 30 mM and 15 mM L-Asp and L-Glu, probably act to enhance Cip uptake by modulating the outer membranes permeability, whilst 15 mM and 7.5 mM are more effective in modulating the membrane permeability of Gram-positive bacteria.

A striking observation is that despite amino acids substantially effecting the growth of *S*. *aureus* in a concentration dependent manner, they seem to exert no or minimal effect on the growth of *P*. *aeruginosa*. What makes this even more interesting is that despite their lack of inhibitory effect on *P*. *aeruginosa*, when they are combined with the drug, a substantial enhancement in antimicrobial activity is observed. This is observed with both TC and Cip but is more apparent with TC (S7 and S8 Figs). Either way, this makes it more likely that the role played by amino acids in this is to increase the effectiveness of the drug, rather than exhibit intrinsic antibiotic activity. Again this increases the possibility that the underlying mechanism involves an increased uptake or reduced efflux of the drug. However, the enhanced antimicrobial activity on the growth of *P*. *aeruginosa* by the combinations of the drug with 7.5 mM of either L-Asp or L-Glu, hints towards other mechanisms of enhancing drug effectiveness, independent of those effecting intracellular drug concentrations, to be also involved. This is because the enhanced effectiveness of the drugs, both Cip and TC, was not accompanied by a reduction in EtBr efflux when combined with 7.5 mM of either amino acid. Either way, due to the reasons just discussed (i.e. lack of antimicrobial activity of amino acids on *P*. *aeruginosa* growth), an accumulative effect of antimicrobial activity of the amino acids and the drug is highly unlikely to be the cause of the enhanced antimicrobial activity observed when they are combined.

As mentioned earlier, some of the adjuvants used and even those under research are probably liable to resistance. Considering both the results obtained with Cip and TC, it seems the potential mechanisms leading to enhanced antimicrobial activity here may be very non-specific. These mechanisms can include an increase in drug uptake, and prevention of efflux by acidic amino acids, however will need to be elucidated through further experimentation. It is likely that identical mechanisms may be involved in the case of both Cip and TC, and some of these will be briefly hypothesized later (section 3.2) in the context of Cip. Nevertheless, if proven, it is hoped that the non-specificity of the mechanisms would make it highly unlikely for resistance mechanisms to evolve against these amino acids. Clinical application of such non-specific mechanisms however will need exploration and appropriate development and control in order to avoid potential toxicity issues.

### 3.1 An assumption explored

Theoretically, one would expect Cip to have competed with EtBr for being effluxed, and thus influenced the pattern of EtBr efflux observed here. However, this cannot be confirmed until the pattern of Cip accumulation is itself measured and analysed alongside that of EtBr to determine if there is a correlation between the two. If this was proved, the method presented in this paper may open doors to the prediction of the efflux of a drug in question by analyzing the pattern of efflux of a co-administered dye. In this sub-section we wish to touch upon how such data could be analyzed and predict for efflux of the drug. For this, we will use the data from efflux assays presented in this paper, where efflux of EtBr was determined in the presence of Cip. To demonstrate this, we assume here that the patter of efflux of EtBr predicts for the efflux and or accumulation of Cip.

With this assumption in mind, and observing a rise in intracellular EtBr, we can assume that Cip levels within the bacteria are also rising; the rise in intracellular level of Cip competes with EtBr for efflux and thus increase the amount of EtBr which is retained. Although in reality, the relationship between EtBr and Cip, if present, is likely to be more complex. This complexity is demonstrated in one of the cases (Fig 2) where the lack of Cip causes intracellular EtBr to reach higher values than in the presence of Cip. This is surprising since the presence of Cip is expected to compete with the efflux of EtBr, which would be expected to result in raising the intracellular levels of the latter. Nevertheless, this hints towards a mechanism which may be efflux independent and instead may involve an increase in the rate of Cip and EtBr uptake due to amino acids through mutual transporter or another mechanism. In such a case, presence of Cip probably competes with EtBr for uptake, potentially through amino acid channels as discussed later. Thus the higher intracellular EtBr without Cip seen in Fig 2 may be due to no competition in its uptake, due to the lack of Cip. Following the rise in EtBr, the subsequent reduction in fluorescence observed with *S*. *aureus* (Figs 1 and 2) and partly with *P*. *aeruginosa* (Fig 5) can be explained by cell death secondary to raised intracellular Cip levels.

The process of predicting drug efflux or accumulation using a secondary dye may be made complicated by the possibility of cell death, as is demonstrated in this example. Interestingly, the growth curves for *S*. *aureus* give an indication as to why 30 mM L-Asp or L-L-Glu combined with Cip did not follow the concentration dependent efflux pattern observed with other amino acid concentrations whilst also giving insight into the steep reduction in fluorescence intensity observed with 15 mM and 7.5 mM these amino acids during efflux experiments. Growth curve data clearly shows that when 15 mM and 7.5 mM L-Asp or L-Glu are combined with Cip, *S*. *aureus*, growth is further hindered. Keeping in mind that these combinations also cause an initial surge of EtBr and therefore (as we are assuming here) Cip within the bacteria, it is possible that bactericidal activity of the increased Cip within the cells causes cell death [33]. The resulting cell death would subsequently enable EtBr to leak out of cells, and since EtBr has differential intracellular and extracellular fluorescence, a great reduction in fluorescence intensity is observed [17]. This is also likely to be the reason for the efflux pattern obtained when 15 mM and 7.5 mM of the amino acids were combined with TC (S1, S2, S5 and S6 Figs). In other words, the reduction in fluorescence intensity observed with Cip or TC combined with 15 mM or 7.5 mM of either amino acid may not be due to EtBr efflux, but rather due to a reduction in intracellular EtBr secondary to cell death. Since when Cip is combined with 30 mM L-Asp or L-Glu, there is no reduction in *S*. *aureus* growth, EtBr does not leak out and hence no drastic reduction in fluorescence is observed, explaining why the efflux pattern observed with these combinations (Cip with 30 mM L-Asp or L-Glu) present as outlier to the concentration dependent pattern observed with other concentrations.

Another interesting observation was that the pattern of efflux, though different in the two bacteria used, was similar irrespective of the concentration of Cip used. This was the case with different Cip concentrations studied in both *S*. *aureus* and *P*. *aeruginosa*. The observation indicates that the concentration of Cip used did not affect the efflux of EtBr. Yet it is also clear that the presence of Cip does indeed effect the efflux of EtBr, as evident from the clear difference in efflux pattern between 0 mM of any amino acid used as compared to the ‘glu’ control; only difference between the two being the presence or absence of Cip, respectively. A potential explanation for this apparent discrepancy may be that the concentration of EtBr (~114 μM) is far greater than the concentrations of Cip used, potentially rendering negligible the difference between the Cip concentrations used. All this also holds true for TC (S1, S2, S5 and S6 Figs), again reiterating that amino acids potentially use a drug independent mechanism of enhancing accumulation. This shows that the development of a method which predicts drug accumulation or efflux using a secondary dye, the concentration of dye used will need to be carefully controlled.

### 3.2 Enhanced ciprofloxacin accumulation

Mechanisms which may be resulting in the enhanced Cip accumulation within *S*. *aureus* and *P*. *aeruginosa* may involve increase uptake, ion-pairing between Cip and the amino acids or inhibition of efflux by the amino acids. These are discussed in this section, the confirmation of which would require further studies.

#### 3.2.1 Increased uptake through porins

Due to the highly selective nature of the outer membrane of Gram-negative bacteria, antibiotics usually utilise porins found in this membrane to penetrate within the cell [14]. OprF is a major outer membrane protein of *P*. *aeruginosa* [34]. OprF has been suggested to allow antibiotic penetration, whilst showing preference to cations [35–37]. Along with this, Cip is known to utilise porins too [38]. Since in the presence of acidic/anionic amino acids, a major proportion of Cip is likely to have been present in the cationic form, thus it may be that this charge eases the passage of Cip through porins like OprF, penetrating through the selective outer membrane and reaching the periplasm. Here the comparatively higher pH shifts the equilibrium towards more neutral molecules of Cip which are than able to passively diffuse through the inner membrane and reach the cytoplasm to carry out their function. Along with this ion-pairing between acidic amino acids and Cip may be resulting in Cip uptake too.

#### 3.2.2 Ion-pairing between acidic amino acid and ciprofloxacin

Amino acid channels such as ATB(0,+) are able to transport not only amino acids, but also prodrugs formed by reactions such as esterification of a drug with the amino acids [39,40]. Interestingly bacteria also express transporters that are able to carry out a similar function. Transporters belonging to the MFS and ABC-binding cassette superfamilies have been suggested to transport amino acids and their derivatives [41,42]. Thus, since in the given Cip and amino acid concentrations, both exist not only as neutral species but also as oppositely charged species, ion-pairs are likely to form between the cationic Cip and the anionic acidic amino acids, conferring the newly formed species the ability to be recognised and taken up through these amino acid transporter channels.

#### 3.2.3 Amino acids inhibit ciprofloxacin efflux in *S*. *aureus*

Majority of the efflux pumps present in *S*. *aureus* belong to the MFS superfamily [11]. Interestingly, it has been shown that transporters such as those belonging to the MFS superfamily, which function by substance/H^+^ symport, can be inhibited by acidic pH [43]. This is because the acidic pH probably prevents deprotonation of the symporter thus preventing any further transport [43]. This provides an explanation as to why the pattern of Cip and TC accumulation caused by amino acids is similar, and highlights that non-specific mechanisms are probably responsible. It may be that the acidic pH of the surrounding environment caused by the L-Asp and L-Glu prevents deprotonation of the symporter, rendering it inhibited.

#### 3.2.4 Amino acids based Mg^2+^ and Ca^2+^ chelation

**Fig 13:**
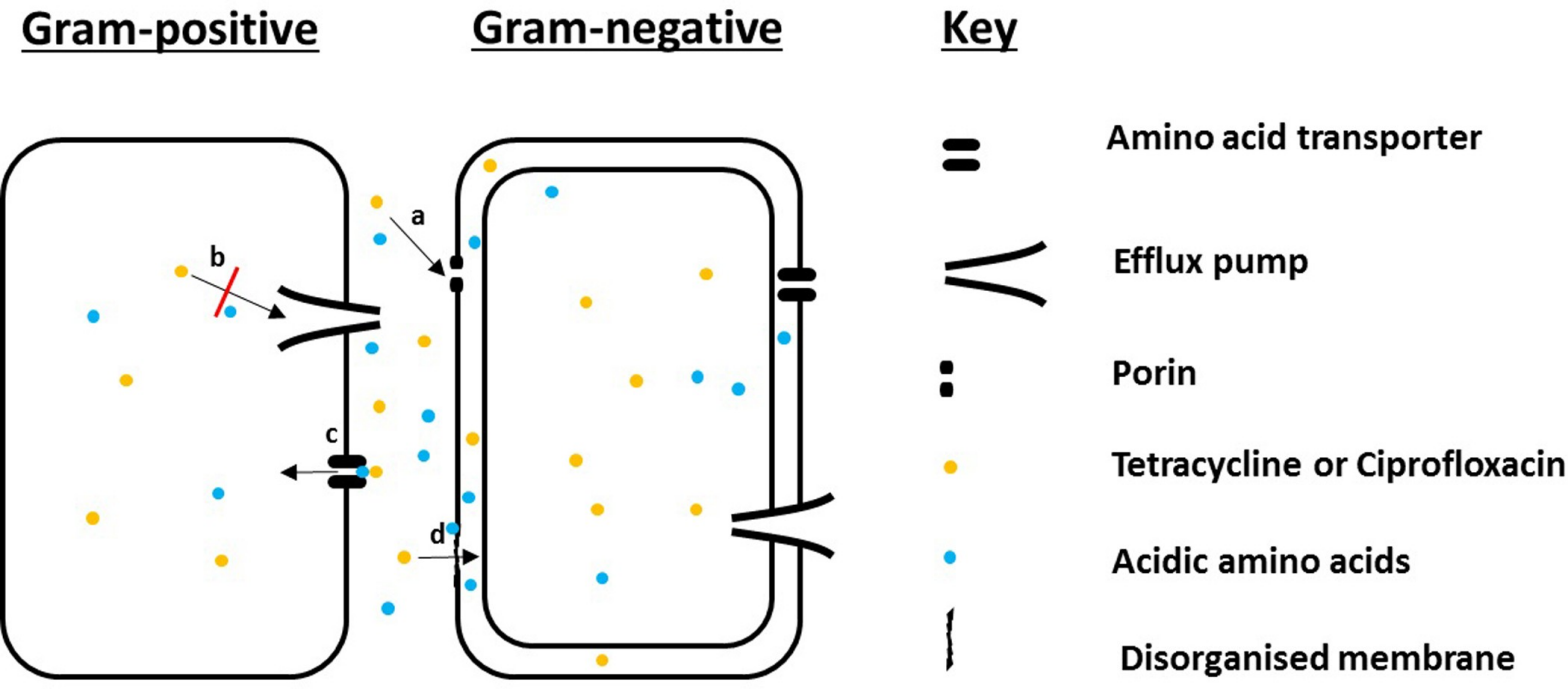
Diagram depicting possible mechanisms utilised by acidic amino acids in enhancing TC and Cip accumulation within both Gram-positive *S*. *aureus* (single membrane) and Gram-negative *P*. *aeruginosa* (two membranes). These mechanisms include a) charge triggered permeation through porins, b) efflux pump inhibition, c) permeation as ion-pairs through amino acid transporters and d) disorganisation of the outer membrane via chelation of otherwise stabilising cations such as Mg^2+^ and Ca^2+^ by acidic amino acids.

Another possible mechanism relates to the ability of amino acids to act as chelating agents. Ethylenediaminetetraacetic acid (EDTA), an anionic substance has been well studied for its ability to chelate cations like Ca^2+^ and Mg^2+^, leading to outer membrane disorganisation, consequently resulting in enhanced permeability of otherwise hard to permeate drugs. These cations play a crucial role in outer membrane integrity by binding to adjacent lipopolysaccharide molecules. Without this, the negative charges present on lipopolysaccharide molecules found on the outer leaflet would cause repulsion between them, resulting in loss of integrity for the outer membrane [44]. Thus Ca^2+^ and Mg^2+^ act by neutralising the negatively charged lipopolysaccharides, maintaining outer leaflet and thus outer membrane stability [44]. Hence like EDTA, here it is proposed that acidic amino acids, a substantial proportion of which would be present as anions, act as Ca^2+^ and Mg^2+^ chelators. This exposes the negative charges present on the lipopolysaccharide molecules, causing repulsion between them. With repulsion between its constituents, the outer membrane loses stability and becomes permeable to Cip and TC, presenting as the enhanced accumulation of these drugs observed in *P*. *aeruginosa*. Supporting this hypothesis is that amino acids, aspartic acid and glutamic acid, are known to act as chelators of metals including Ca^2+^ and Mg^2+^ [45]. The above proposed mechanisms are depicted in Fig 13.

## 4. Materials and methods

### 4.1 Materials

Ciprofloxacin 98%, ethidium bromide 10 mg/ml and tryptone soya broth (TSB) was obtained from Fisher Scientific. L-Aspartic acid 98+% (T), L-glutamic acid 99% (N), D-(+)-glucose 99.5% GC and phosphate buffered saline tablets were from Sigma Aldrich. KCl (Sigma Aldrich; Dorset, England), MgCl_2_ and chloroform (Fisher Scientific; Loughborough, England) were gifted by technical staff (Faculty of Science and Engineering, University of Wolverhampton). *S*. *aureus* strain NCTC 8325 and *P*. *aeruginosa* strain PAO1 was gifted by Professor Peter A. Lambert (School of Life and Health Science, Aston University). TC-treated, flat bottomed 96-well plates were purchased from VWR.

### 4.2 Determining the minimum inhibitory concentrations and minimum bactericidal concentrations of amino acids and ciprofloxacin

Minimum inhibitory concentrations (MICs) and minimum bactericidal concentrations (MBCs) of L-Asp, L-Glu and Cip on *S*. *aureus* and *P*. *aeruginosa* were determined using a method adjusted from literature [46]. To do this, overnight cultures of *S*. *aureus* and *P*. *aeruginosa* were grown in TSB and adjusted to 0.1 optical density (O.D.). This corresponds to a CFU/ml of 1 x 10^8^ for S. aureus and 1 x 10^9^ to 1 x 10^10^ for P. aeruginosa. Each bacterium was then incubated with two-fold serial dilutions of L-Asp, L-Glu or Cip. The concentrations of amino acids used ranged from 30 mM to 0.23 mM whilst the concentration of Cip used ranged from 135.81 μM to 2.12 μM. Following this, the 96-well plates were incubated in a 37°C incubator overnight. MICs were taken to be the lowest concentrations with no visible growth (turbidity); generally, one two-fold dilution below MBCs. To determine MBCs, concentrations which showed no visible growth (turbidity) were plated on TSA agar plates and incubated at 37°C overnight. MBCs were taken to be the lowest concentrations which resulted in no colony formation.

### 4.3 EtBr accumulation assay

The accumulation assay used here was adopted from one previously described [18]. To bring to logarithmic phase, 10 ml of the overnight culture was added to 20ml of fresh TSB and incubated for one hour at 37°C in an orbital shaking incubator. To deprive the cells of energy, the culture was then centrifuged at 2860 g for 20 minutes and the supernatant was discarded. The cell pellet was resuspended in 30 ml 1 mM MgCl_2_ in 0.01 M PBS. The centrifugation step was repeated following which the pellet was resuspended in the same buffer. The cell suspension was split into 4 equal volumes (2.5 ml each) and to each quarter, 2.5 ml of double the working concentration of the drug (made up in the same buffer) along with 250 μl of 1 mg/ml EtBr was added. For example, if the working concentration is 1 mM Cip, add 2.5 ml of 2 mM Cip along with 250 μl of EtBr. Aliquot 100 μl per well in 96 well plate. This sets up the first 60 min of the experiment, where the lack of energy enables maximum accumulation of Cip and EtBr within cells. For this, fluorescence was observed every 5 minutes for 60 min (room temperature) at an excitation wavelength of 510 nm and an emission wavelength of 595 nm.

To study the effect of amino acids on the accumulation or efflux of EtBr, 100 μl of amino acid concentrations ranging from 30 mM to 0.23 mM made up in 1 mM MgCl_2_, 0.01 M PBS and 100 mM glucose (to provide energy) was added to wells as required. Next 20 μl of 1M KCl was added to each well. Controls included no drug but with maximum AA concentration, no glucose with no AA but with maximum drug concentration, no AA, no glucose with no drug or AA and glucose with no drug or AA.

### 4.4 Growth curve susceptibility assay

Growth curves of L-Asp, L-Glu and Cip were evaluated for *S*. *aureus* and *P*. *aeruginosa* in the presence of the combinations used in Cip accumulation assays. To do this, overnight cultures of *S*. *aureus* and *P*. *aeruginosa* were grown in TSB. Solutions with double the working concentrations were prepared for the amino acids, Cip and their combinations in TSB. 100 μL of these were then incubated with 100 μL of the culture in wells of 96 well plates, in such a way that the resulting cell suspension consisted of the desired working concentrations of the test substances along with 0.1 O.D. of bacteria. The plates were then analysed in a shaking and incubated (37°C) plate reader, set to shake the plate and read O.D. (600 nm) of each well every half an hour.

### 4.5 Pyocyanin quantification

After the susceptibility assay, 96 well plates were incubated in the fridge until Pyocyanin was quantified using a previously described method [47]. Briefly, cell-free supernatant was obtained by centrifugation at 3220 g for 10 minutes. 100 μl of chloroform was added to 150 μl of the supernatant and vigorously vortexed. The samples were then centrifuged at 2996 g for 5 minutes and the supernatant was discarded. To the remaining bottom (chloroform) layer, 60 μl of 0.2 M HCl was added, followed by vigorous vortexing. The samples were again centrifuged at 2996 g for 5 minutes and the top layer was transferred to a 96 well plate. After all the samples were processed in this manner, the 96 well plate was read using a plate reader at 520 nm.

### 4.6 Pyoverdin quantification

After susceptibility testing as described above, the 96 well plates were kept in the fridge until pyoverdin was quantified. Briefly, the plates were centrifuged at 3220 g for 10 min. Cell-free supernatant was transferred to Greiner black bottom plates and the concentration of pyoverdin was quantified using a fluorescence spectrophotometer at an excitation wavelength of 395 nm and an emission wavelength of 470 nm. The results were plotted relative to control.

### 4.7 Statistical analysis

Statistical analysis was conducted using GraphPad Prism. Initially one way ANOVA was carried out to look for overall significance within the data. This was followed by the uncorrected Fisher’s LSD test, to see where the significant difference lies. P values below 0.05 for the results were taken as statistically significant. All data presented for growth and pigment production experiments is the mean of at least three biological replicates from independent bacterial cultures. Efflux data presented here is a mean of four replicates.

## 5. Conclusions

In conclusion, acidic amino acids have been shown in this study to act as adjuvants able to overcome resistance to Cip in both *S*. *aureus* and *P*. *aeruginosa* by enhancing antimicrobial activity and thus inhibiting the growth of bacteria. Likewise, synergistic reduction in pigment production by *P*. *aeruginosa* was also observed by combining the amino acids with the drug. This appears to result from the ability of L-Asp and L-Glu to modulate efflux mechanism of these bacteria, as evident from the reduced efflux and increased accumulation of EtBr. The amino acids probably function through multiple mechanisms. Whilst the results suggest that the mechanism utilised are not drug-dependent, they probably depend on whether the bacteria in question is Gram-negative or Gram-positive. These findings may hold a key to strategies for overcoming the ever increasing AMR and it is hoped that the potential underlying nonspecific mechanisms will make it substantially rarer for resistance to emerge.

## Supporting information

S1 Fig Efflux of EtBr by *S*. *aureus* in the presence of different concentrations of L-Asp (30 mM to 0.23 mM). A) 3.52 μM TC B) 1.76 μM TC C) 0.88 μM TC D) 0.44 μM TC. In the legend, numbers 0 to 30 represent amino acid concentrations in combination with respective TC concentration. Firstly, the dashed line represents end of the first part of experiment where Cip and EtBr was allowed to accumulate within energy deprived cells and secondly the start of accumulation in the presence of the amino acid, with or without energy; n = 4.
(TIF)

S2 Fig Efflux of EtBr by *S*. *aureus* in the presence of different concentrations of L-Glu (30 mM to 0.23 mM). A) 3.52 μM TC B) 1.76 μM TC C) 0.88 μM TC D) 0.44 μM TC. In the legend, numbers 0 to 30 represent amino acid concentrations in combination with respective TC concentration. Firstly, the dashed line represents end of the first part of experiment where Cip and EtBr was allowed to accumulate within energy deprived cells and secondly the start of accumulation in the presence of the amino acid, with or without energy; n = 4.
(TIF)

S3 Fig S. aureus growth curves with A) 30, 15 and 7.5 mM L-Asp amino acid on its own, B) with 3.52, 1.76, 0.88 and 0.44 μM TC on its own, C) 30 mM L-Asp combined with 3.52, 1.76, 0.88 and 0.44 μM TC, D) 15 mM L-Asp combined with 3.52, 1.76, 0.88 and 0.44 μM TC and E) 7.5 mM L-Asp combined with 3.52, 1.76, 0.88 and 0.44 μM TC; n = 3.
(TIF)

S4 Fig S. aureus growth curves with A) 30, 15 and 7.5 mM L-Glu amino acid on its own, B) with 3.52, 1.76, 0.88 and 0.44 μM TC on its own, C) 30 mM L-Glu combined with 3.52, 1.76, 0.88 and 0.44 μM TC, D) 15 mM L-Glu combined with 3.52, 1.76, 0.88 and 0.44 μM TC and E) 7.5 mM L-Glu combined with 3.52, 1.76, 0.88 and 0.44 μM TC; n = 3.
(TIF)

S5 Fig Efflux of EtBr by *P*. *aeruginosa* in the presence of different concentrations of L-Asp (30 mM to 0.23 mM). A) 112.50 μM TC B) 56.25 μM TC C) 28.13 μM TC D) 14.06 μM TC. In the legend, numbers 0 to 30 represent amino acid concentrations in combination with respective TC concentration. Firstly, the dashed line represents end of the first part of experiment where Cip and EtBr was allowed to accumulate within energy deprived cells and secondly the start of accumulation in the presence of the amino acid, with or without energy; n = 4.
(TIF)

S6 Fig Efflux of EtBr by *P*. *aeruginosa* in the presence of different concentrations of L-Glu (30 mM to 0.23 mM). A) 112.50 μM TC B) 56.25 μM TC C) 28.13 μM TC D) 14.06 μM TC. In the legend, numbers 0 to 30 represent amino acid concentrations in combination with respective TC concentration. Firstly, the dashed line represents end of the first part of experiment where Cip and EtBr was allowed to accumulate within energy deprived cells and secondly the start of accumulation in the presence of the amino acid, with or without energy; n = 4.
(TIF)

S7 Fig P. aeruginosa growth curves with A) 30, 15 and 7.5 mM L-Asp amino acid on its own, B) with 112.50, 56.25, 28.13 and 14.06 μM TC on its own, C) 30 mM L-Asp combined with 112.50, 56.25, 28.13 and 14.06 μM TC, D) 15 mM L-Asp combined with 112.50, 56.25, 28.13 and 14.06 μM TC and E) 7.5 mM L-Asp combined with 112.50, 56.25, 28.13 and 14.06 μM TC; n = 3.
(TIF)

S8 Fig P. aeruginosa growth curves with A) 30, 15 and 7.5 mM L-Glu amino acid on its own, B) with 112.50, 56.25, 28.13 and 14.06 μM TC on its own, C) 30 mM L-Glu combined with 112.50, 56.25, 28.13 and 14.06 μM TC, D) 15 mM L-Glu combined with 112.50, 56.25, 28.13 and 14.06 μM TC and E) 7.5 mM L-Glu combined with 112.50, 56.25, 28.13 and 14.06 μM TC; n = 3.
(TIF)

S9 Fig Effect of L-Asp, TC and L-Asp and TC combinations on the production of pyocyanin by *P*. *aeruginosa*. Y-axis shows concentration of pyocyanin in normalized units when treated with 30, 15 and 7.5 mM L-Asp, 112.50, 56.25, 28.13 and 14.06 μM TC and their combinations. Significant results are indicated by * or **, where * represent a significant reduction in pigment production by the amino acid or TC compared to control and ** represents a significant reduction in pigment production by combinations (AA + TC) compared to both of the individual corresponding components (p < 0.05 was taken as significant); n = 3.
(TIF)

S10 Fig Effect of L-Glu, TC and L-Glu and TC combinations on the production of pyocyanin by *P*. *aeruginosa*. Y-axis shows concentration of pyocyanin in normalized units when treated with 30, 15 and 7.5 mM L-Glu, 112.50, 56.25, 28.13 and 14.06 μM TC and their combinations. Significant results are indicated by * or **, where * represent a significant reduction in pigment production by the amino acid or TC compared to control and ** represents a significant reduction in pigment production by combinations (AA + TC) compared to both of the individual corresponding components (p < 0.05 was taken as significant); n = 3.
(TIF)

S11 Fig Effect of L-Asp, TC and L-Asp and TC combinations on the production of pyoverdine by *P*. *aeruginosa*. Y-axis shows concentration of pyoverdine as a percentage of untreated, when treated with 30, 15 and 7.5 mM L-Asp, 112.50, 56.25, 28.13 and 14.06 μM Cip and their combinations. Significant results are indicated by * or **, where * represent a significant reduction in pigment production by the amino acid or TC compared to control and ** represents a significant reduction in pigment production by combinations (AA + TC) compared to both of the individual corresponding components (p < 0.05 was taken as significant); n = 3.
(TIF)

S12 Fig Effect of L-Glu, TC and L-Glu and TC combinations on the production of pyoverdine by P. aeruginosa. Y-axis shows concentration of pyoverdine as a percentage of untreated, when treated with 30, 15 and 7.5 mM L-Glu, 112.50, 56.25, 28.13 and 14.06 μM Cip and their combinations. Significant results are indicated by * or **, where * represent a significant reduction in pigment production by the amino acid or TC compared to control and ** represents a significant reduction in pigment production by combinations (AA + TC) compared to both of the individual corresponding components (p < 0.05 was taken as significant); n = 3.
(TIF)
