# Supplementary figures and images for: Acidic amino acids as counterions of ciprofloxacin: Effect on growth and pigment production in Staphylococcus aureus NCTC 8325 and Pseudomonas aeruginosa PAO1
Source: PLoS One. 2021 Apr 29;16(4):e0250705. doi: 10.1371/journal.pone.0250705 (PMC8084218; doi:10.1371/journal.pone.0250705)

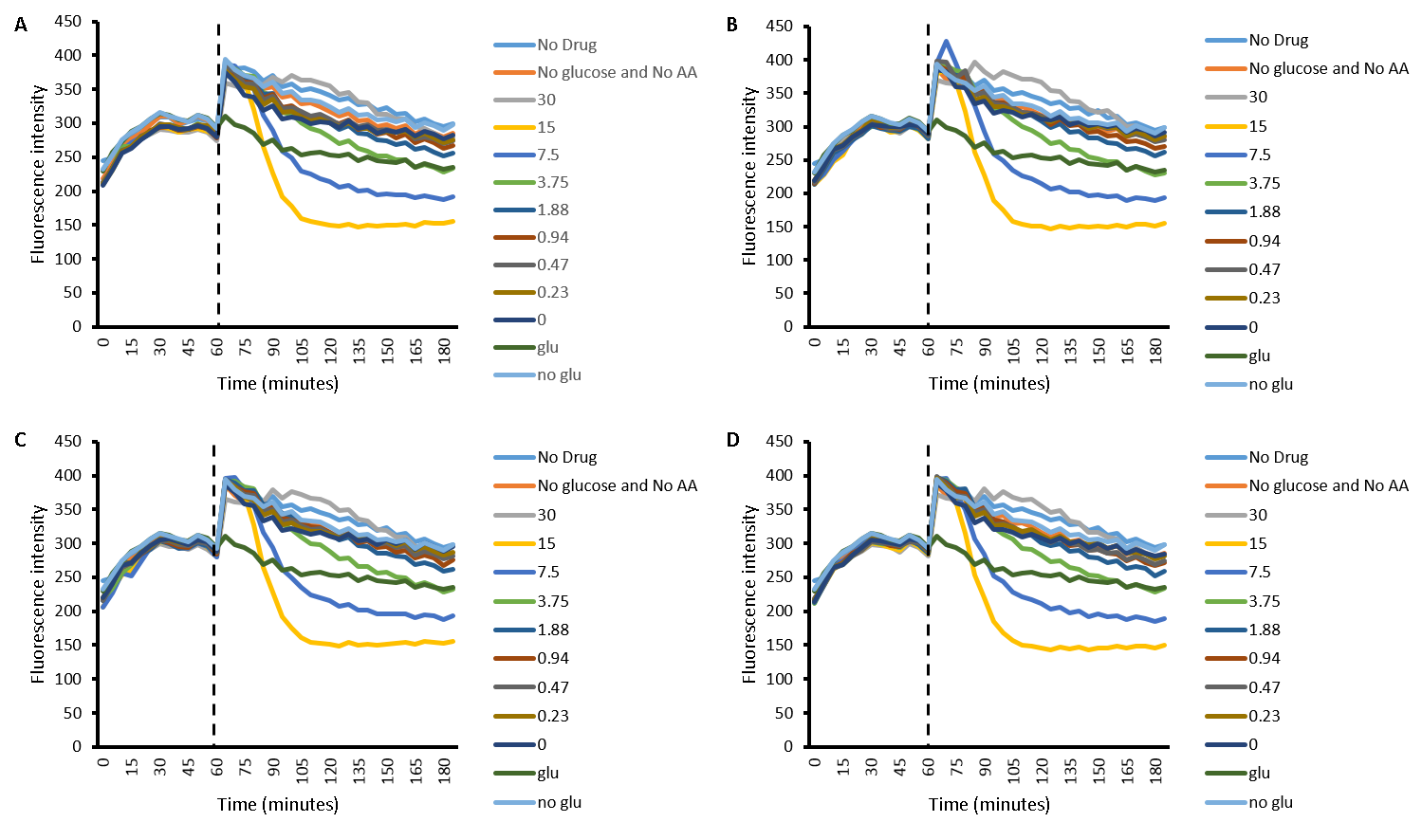

Supplement: S1 Fig — A) 3.52 μM TC B) 1.76 μM TC C) 0.88 μM TC D) 0.44 μM TC. In the legend, numbers 0 to 30 represent amino acid concentrations in combination with respective TC concentration. Firstly, the dashed line represents end of the first part of experiment where Cip and EtBr was allowed to accumulate within energy deprived cells and secondly the start of accumulation in the presence of the amino acid, with or without energy; n = 4. (TIF) [file pone.0250705.s001.tif]

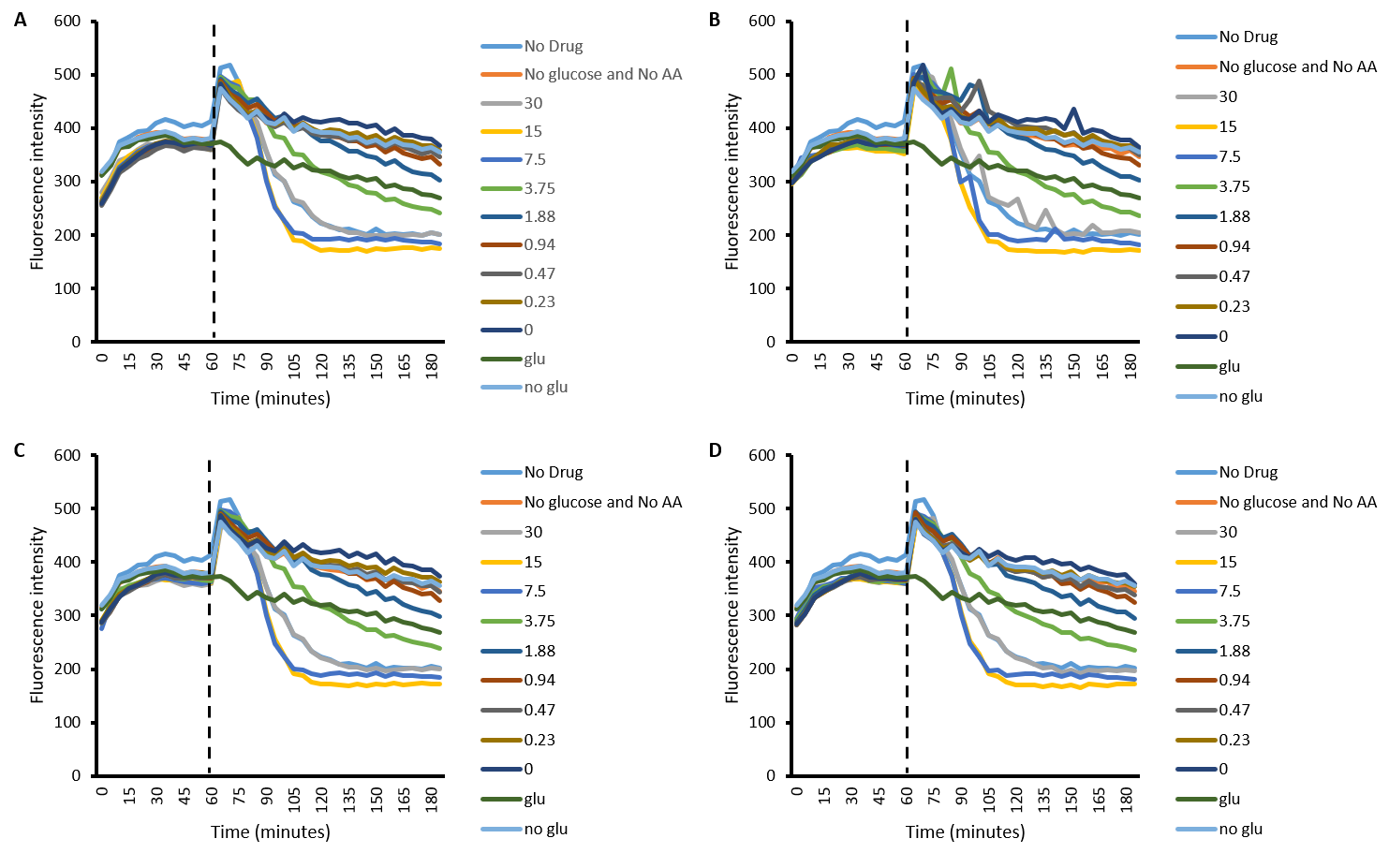

Supplement: S2 Fig — A) 3.52 μM TC B) 1.76 μM TC C) 0.88 μM TC D) 0.44 μM TC. In the legend, numbers 0 to 30 represent amino acid concentrations in combination with respective TC concentration. Firstly, the dashed line represents end of the first part of experiment where Cip and EtBr was allowed to accumulate within energy deprived cells and secondly the start of accumulation in the presence of the amino acid, with or without energy; n = 4. (TIF) [file pone.0250705.s002.tif]

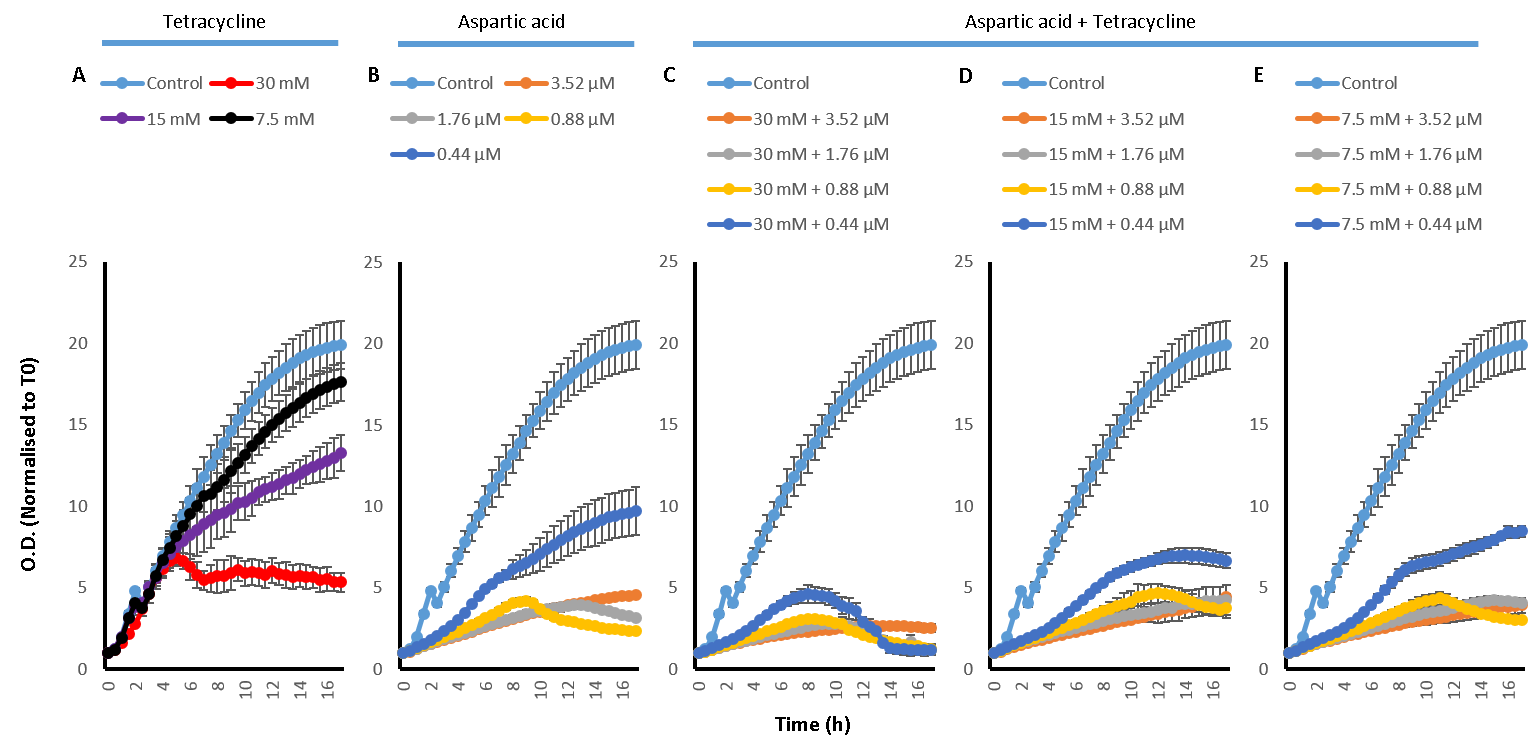

Supplement: S3 Fig — S. aureus growth curves with A) 30, 15 and 7.5 mM L-Asp amino acid on its own, B) with 3.52, 1.76, 0.88 and 0.44 μM TC on its own, C) 30 mM L-Asp combined with 3.52, 1.76, 0.88 and 0.44 μM TC, D) 15 mM L-Asp combined with 3.52, 1.76, 0.88 and 0.44 μM TC and E) 7.5 mM L-Asp combined with 3.52, 1.76, 0.88 and 0.44 μM TC; n = 3. (TIF) [file pone.0250705.s003.tif]

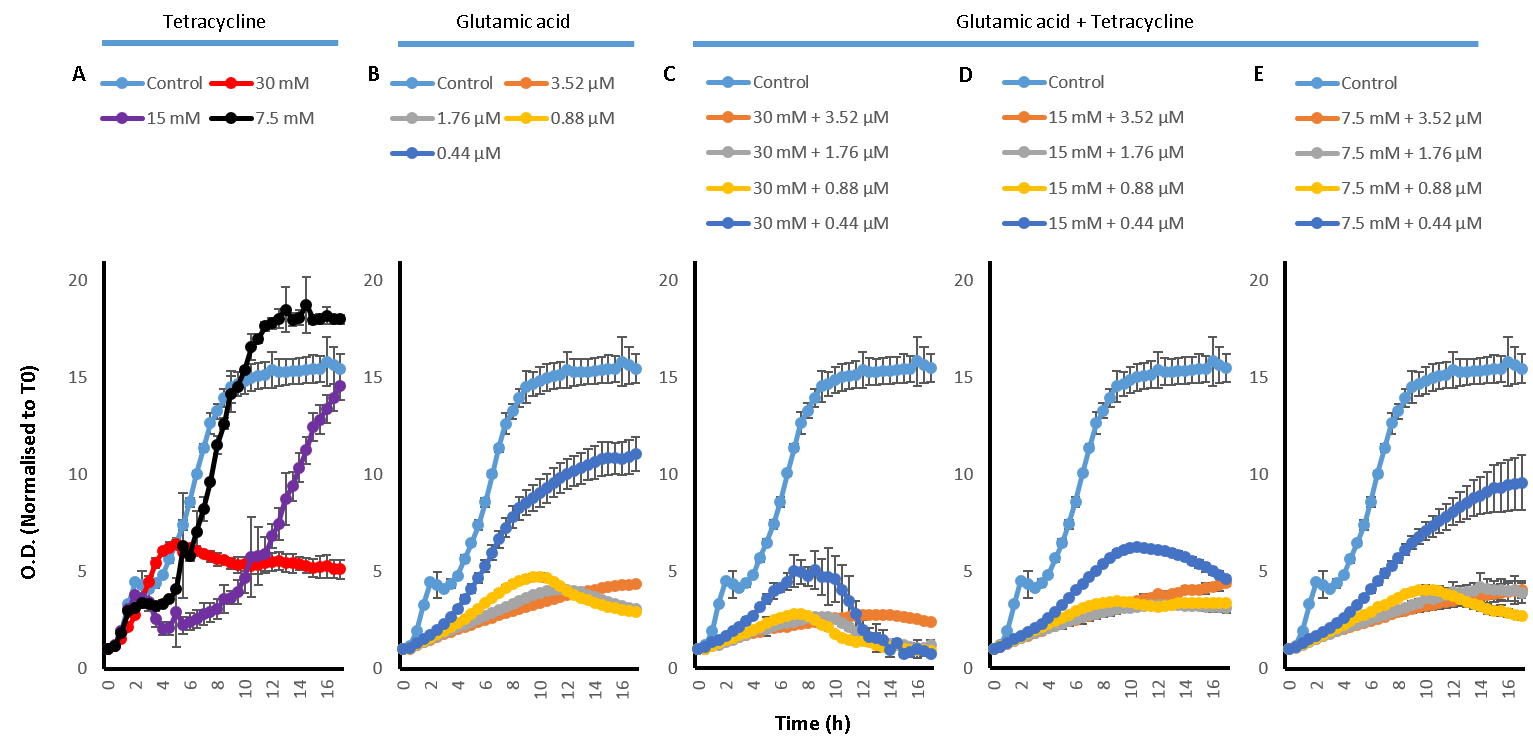

Supplement: S4 Fig — S. aureus growth curves with A) 30, 15 and 7.5 mM L-Glu amino acid on its own, B) with 3.52, 1.76, 0.88 and 0.44 μM TC on its own, C) 30 mM L-Glu combined with 3.52, 1.76, 0.88 and 0.44 μM TC, D) 15 mM L-Glu combined with 3.52, 1.76, 0.88 and 0.44 μM TC and E) 7.5 mM L-Glu combined with 3.52, 1.76, 0.88 and 0.44 μM TC; n = 3. (TIF) [file pone.0250705.s004.tif]

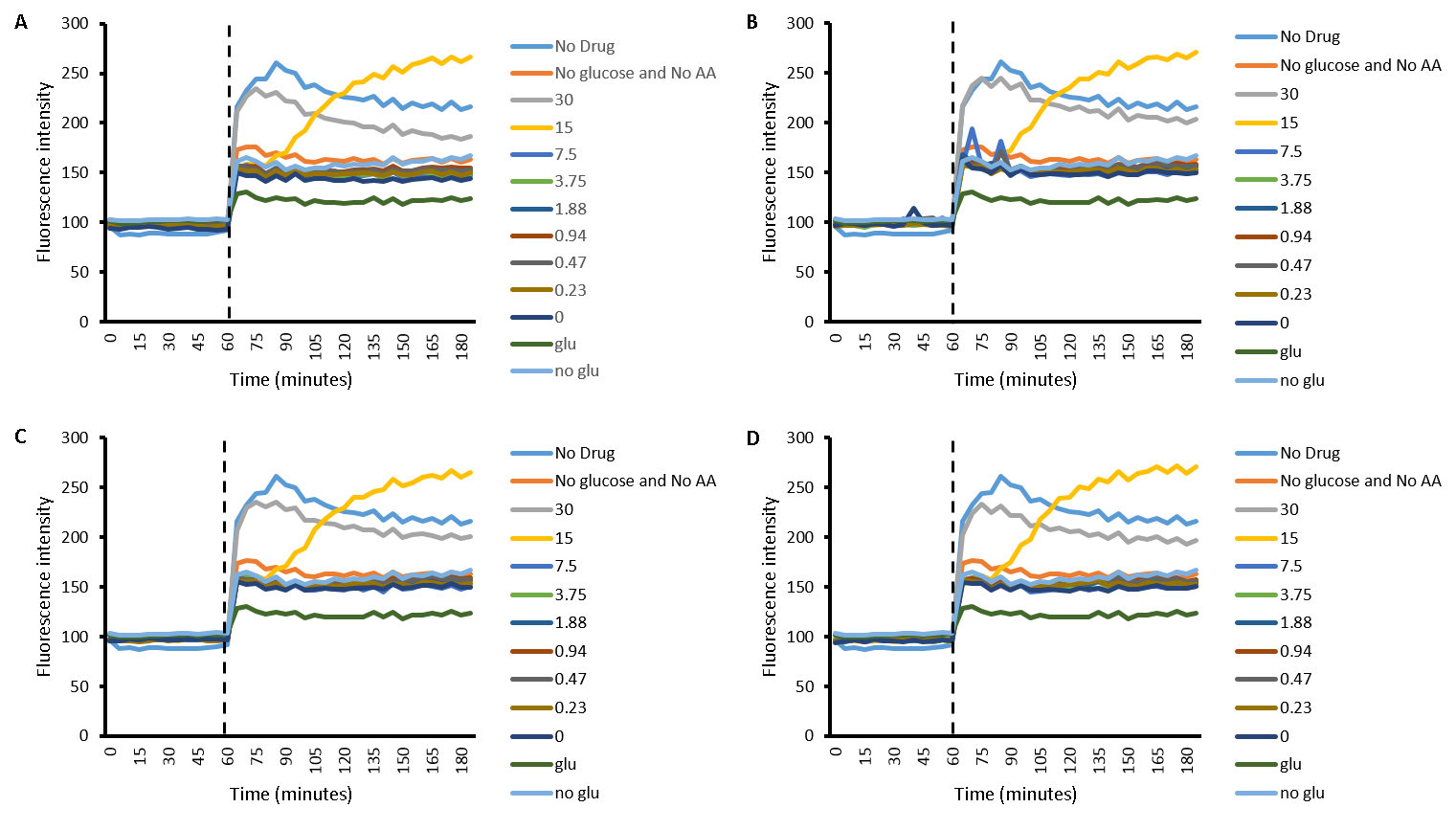

Supplement: S5 Fig — A) 112.50 μM TC B) 56.25 μM TC C) 28.13 μM TC D) 14.06 μM TC. In the legend, numbers 0 to 30 represent amino acid concentrations in combination with respective TC concentration. Firstly, the dashed line represents end of the first part of experiment where Cip and EtBr was allowed to accumulate within energy deprived cells and secondly the start of accumulation in the presence of the amino acid, with or without energy; n = 4. (TIF) [file pone.0250705.s005.tif]

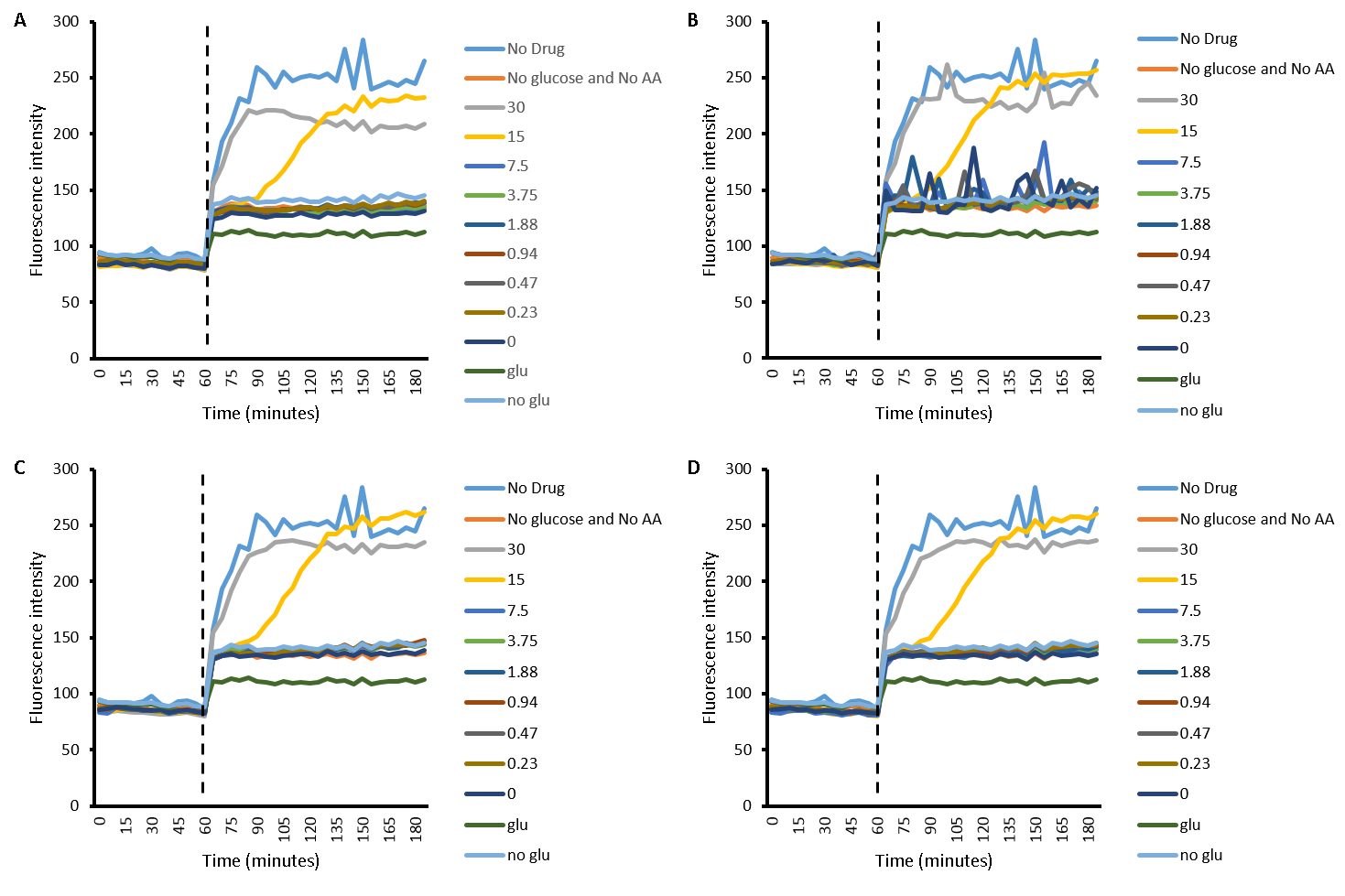

Supplement: S6 Fig — A) 112.50 μM TC B) 56.25 μM TC C) 28.13 μM TC D) 14.06 μM TC. In the legend, numbers 0 to 30 represent amino acid concentrations in combination with respective TC concentration. Firstly, the dashed line represents end of the first part of experiment where Cip and EtBr was allowed to accumulate within energy deprived cells and secondly the start of accumulation in the presence of the amino acid, with or without energy; n = 4. (TIF) [file pone.0250705.s006.tif]

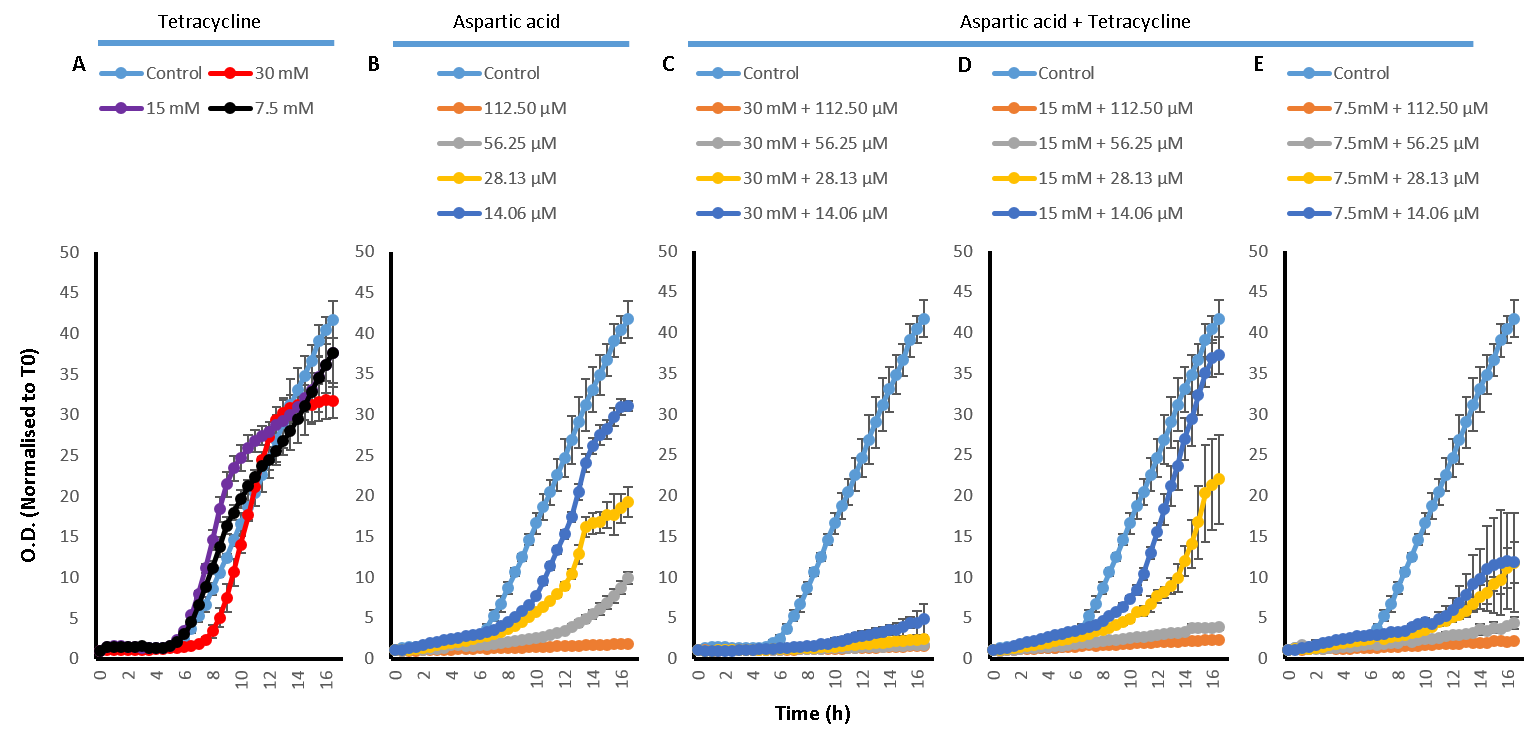

Supplement: S7 Fig — P. aeruginosa growth curves with A) 30, 15 and 7.5 mM L-Asp amino acid on its own, B) with 112.50, 56.25, 28.13 and 14.06 μM TC on its own, C) 30 mM L-Asp combined with 112.50, 56.25, 28.13 and 14.06 μM TC, D) 15 mM L-Asp combined with 112.50, 56.25, 28.13 and 14.06 μM TC and E) 7.5 mM L-Asp combined with 112.50, 56.25, 28.13 and 14.06 μM TC; n = 3. (TIF) [file pone.0250705.s007.tif]

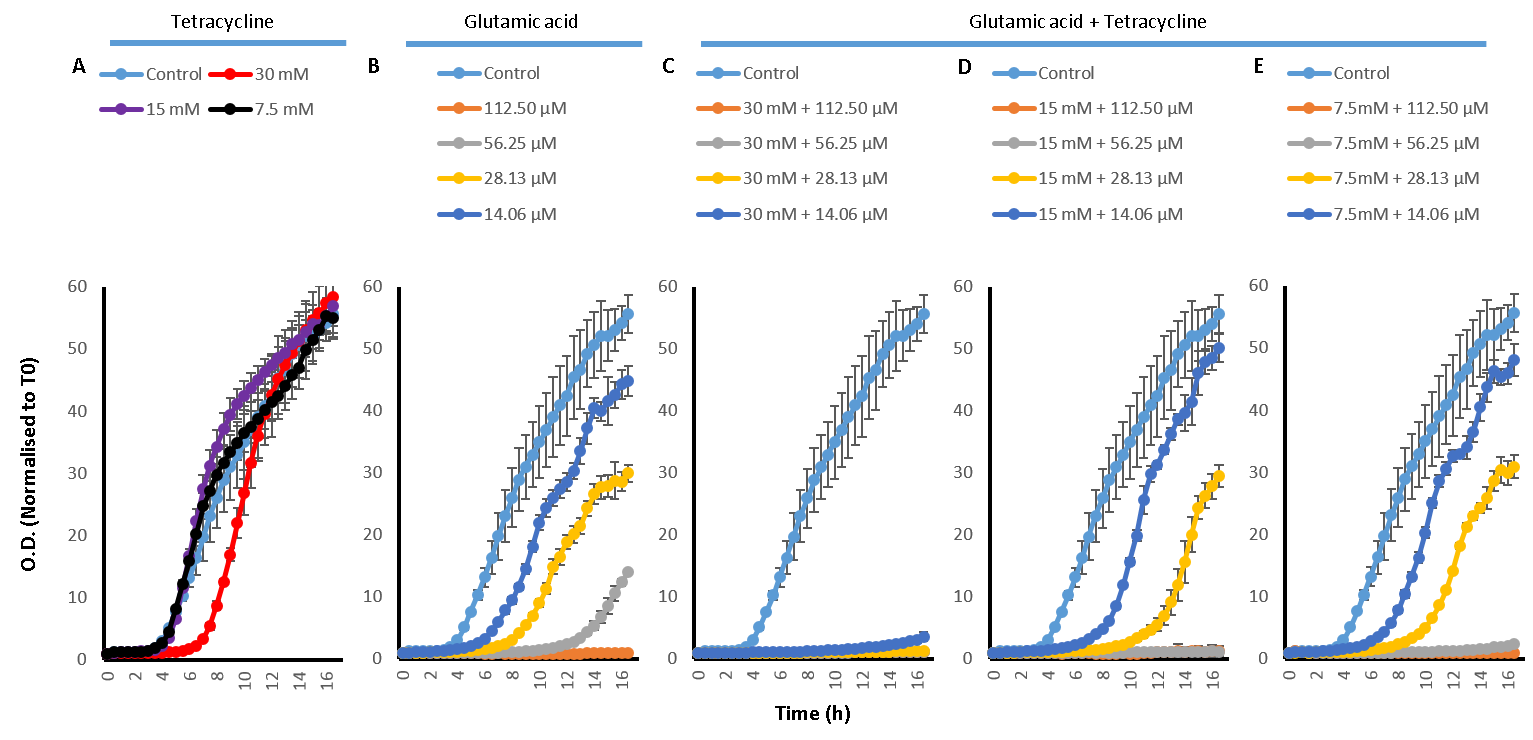

Supplement: S8 Fig — P. aeruginosa growth curves with A) 30, 15 and 7.5 mM L-Glu amino acid on its own, B) with 112.50, 56.25, 28.13 and 14.06 μM TC on its own, C) 30 mM L-Glu combined with 112.50, 56.25, 28.13 and 14.06 μM TC, D) 15 mM L-Glu combined with 112.50, 56.25, 28.13 and 14.06 μM TC and E) 7.5 mM L-Glu combined with 112.50, 56.25, 28.13 and 14.06 μM TC; n = 3. (TIF) [file pone.0250705.s008.tif]

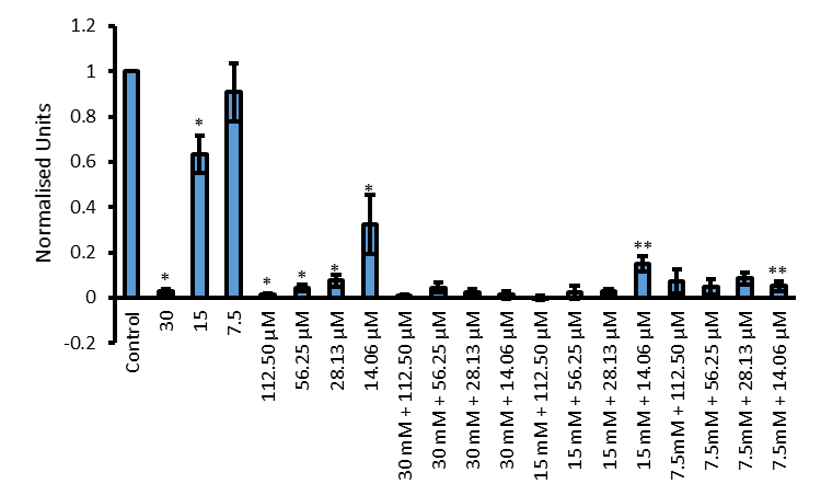

Supplement: S9 Fig — Y-axis shows concentration of pyocyanin in normalized units when treated with 30, 15 and 7.5 mM L-Asp, 112.50, 56.25, 28.13 and 14.06 μM TC and their combinations. Significant results are indicated by * or **, where * represent a significant reduction in pigment production by the amino acid or TC compared to control and ** represents a significant reduction in pigment production by combinations (AA + TC) compared to both of the individual corresponding components (p < 0.05 was taken as significant); n = 3. (TIF) [file pone.0250705.s009.tif]

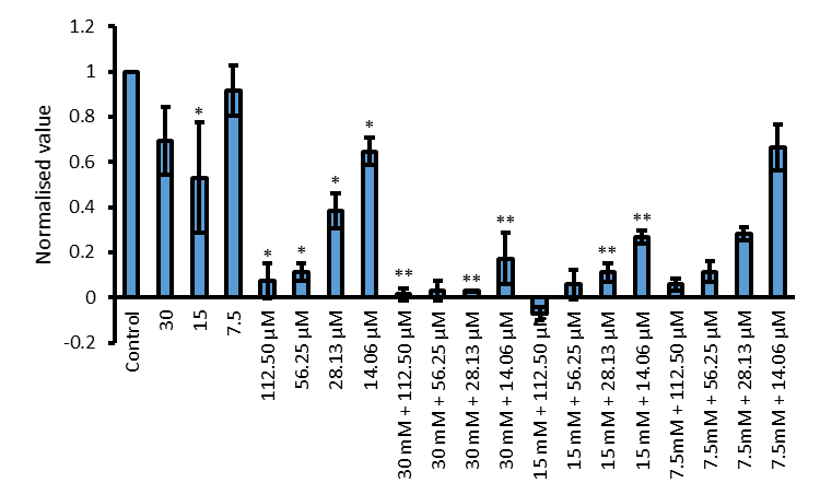

Supplement: S10 Fig — Y-axis shows concentration of pyocyanin in normalized units when treated with 30, 15 and 7.5 mM L-Glu, 112.50, 56.25, 28.13 and 14.06 μM TC and their combinations. Significant results are indicated by * or **, where * represent a significant reduction in pigment production by the amino acid or TC compared to control and ** represents a significant reduction in pigment production by combinations (AA + TC) compared to both of the individual corresponding components (p < 0.05 was taken as significant); n = 3. (TIF) [file pone.0250705.s010.tif]

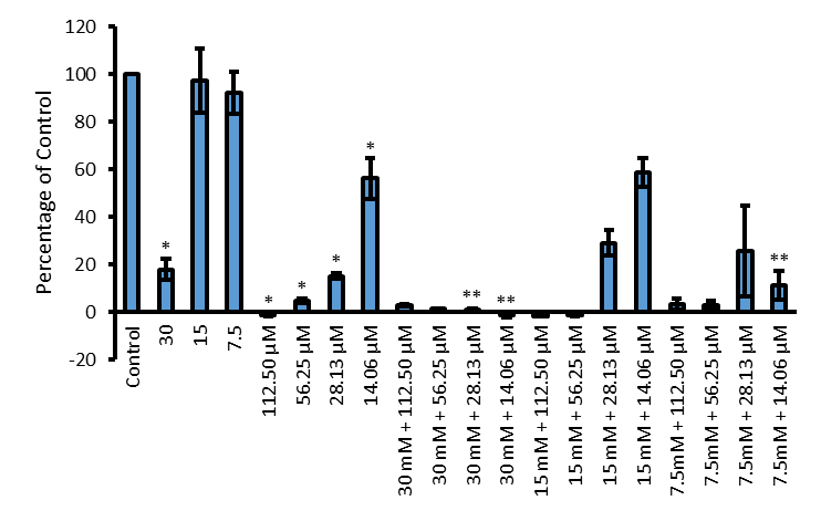

Supplement: S11 Fig — Y-axis shows concentration of pyoverdine as a percentage of untreated, when treated with 30, 15 and 7.5 mM L-Asp, 112.50, 56.25, 28.13 and 14.06 μM Cip and their combinations. Significant results are indicated by * or **, where * represent a significant reduction in pigment production by the amino acid or TC compared to control and ** represents a significant reduction in pigment production by combinations (AA + TC) compared to both of the individual corresponding components (p < 0.05 was taken as significant); n = 3. (TIF) [file pone.0250705.s011.tif]

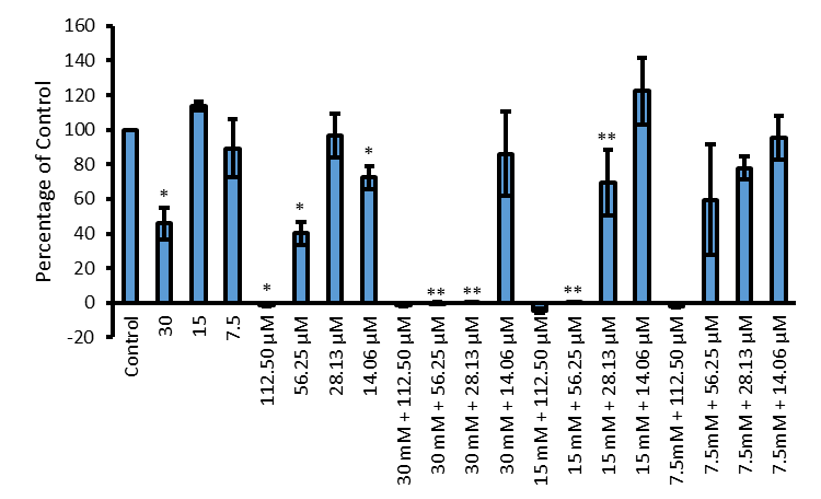

Supplement: S12 Fig — Y-axis shows concentration of pyoverdine as a percentage of untreated, when treated with 30, 15 and 7.5 mM L-Glu, 112.50, 56.25, 28.13 and 14.06 μM Cip and their combinations. Significant results are indicated by * or **, where * represent a significant reduction in pigment production by the amino acid or TC compared to control and ** represents a significant reduction in pigment production by combinations (AA + TC) compared to both of the individual corresponding components (p < 0.05 was taken as significant); n = 3. (TIF) [file pone.0250705.s012.tif]
